# Supplementary figures and images for: Different depths of sedation versus risk of delirium in adult mechanically ventilated patients: A systematic review and meta-analysis
Source: PLoS One. 2020 Jul 16;15(7):e0236014. doi: 10.1371/journal.pone.0236014 (PMC7365415; doi:10.1371/journal.pone.0236014)

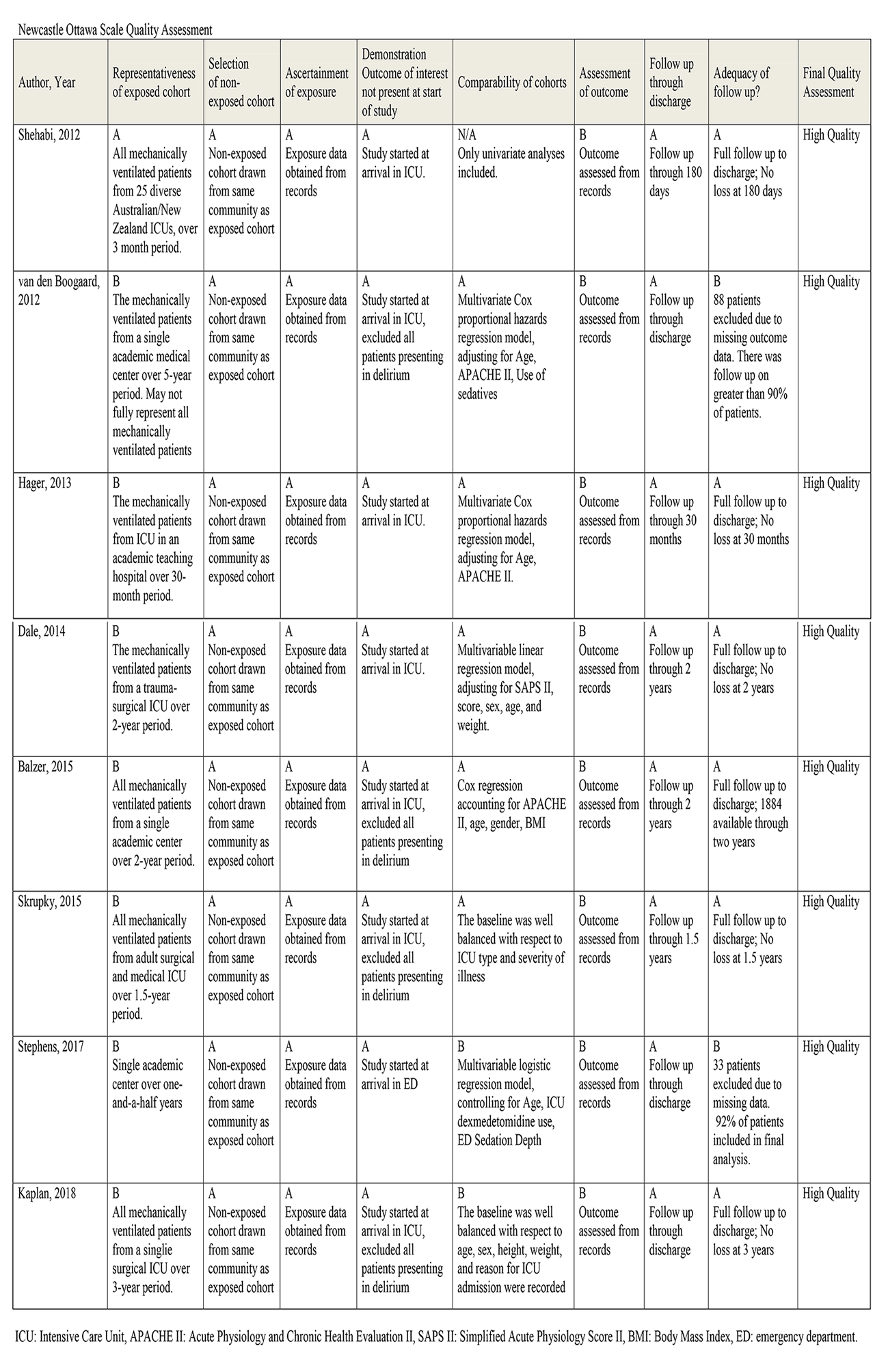

Supplement: S1 Fig — ICU: Intensive Care Unit, APACHE II: Acute Physiology and Chronic Health Evaluation II, SAPS II: Simplified Acute Physiology Score II, BMI: Body Mass Index, ED: emergency department. (TIF) [file pone.0236014.s002.tif]

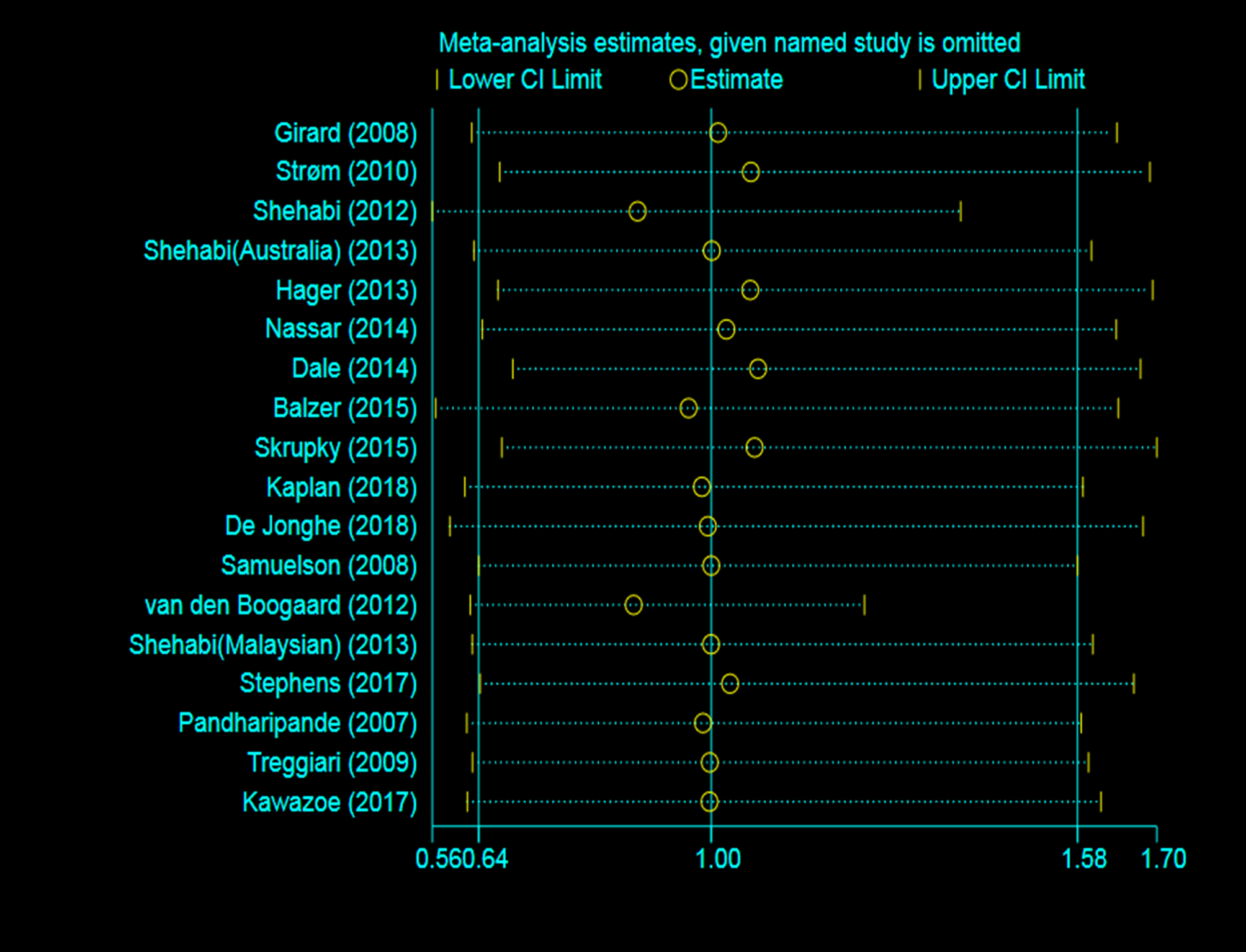

Supplement: S2 Fig — (TIF) [file pone.0236014.s003.tif]
