# Supplementary material for: Different depths of sedation versus risk of delirium in adult mechanically ventilated patients: A systematic review and meta-analysis
Source: PLoS One. 2020 Jul 16;15(7):e0236014. doi: 10.1371/journal.pone.0236014 (PMC7365415; doi:10.1371/journal.pone.0236014)
Supplement: S1 Table — D/L: Deeper/Lighter gourp, BDZ: benzodiazepine (Different BDZ means there was a significant difference in BDZ dosage between two groups), 1: mg/h, 2: mg/d, 3: mg(cumulative dose) 4: mg/kg/h, 5: mg/kg(cumulative dose), 6: %(% of patient midazolam was given), -: means without BZD or data unavailable. a: APACHEⅡ(Acute Physiology and Chronic Health EvaluationⅡ), b: SAPSⅡ (Simplified Acute Physiology ScoreⅡ), C: SOFA (Sequential Organ Failure Assessment) (DOC) [file pone.0236014.s004.doc]

Benzodiazepine used and patients’ severity of illness in the included studies

| **Author/Year** | **Number**  **(D/L)** | Benzodiazepine use | | | Severity of illness | |
| --- | --- | --- | --- | --- | --- | --- |
|  |  | **Drug** | **Dosage (D/L)** | **P-value** | **Score(D/L)** | **P-value** |
| Pandharipande et al^17^ (2007) | 51/52 | lorazepam | 3 vs 0 ^1^ | ＜0.05 | 27 vs 29 ^a^ | 0.75 |
| Samuelson et al^14^ (2008) | 18/18 | - | - | - | 11 vs 9.5 ^a^ | 0.94 |
| Girard et al^16^ (2008) | 168/167 | Benzodiazepine | 3 vs 2 ^2^ | 0.12 | 26 vs 26.5 ^a^ | ＞0.05 |
| Treggiari et al^15^ (2009) | 64/65 | Benzodiazepine | 53 vs 39 ^3^ | ＜0.05 | 36.5 vs 36.0 ^b^ | ＞0.05 |
| Strøm et al^18^ (2010) | 58/55 | Midazolam | 0.0034 vs 0 ^4^ | ＜0.001 | 26 vs 26 ^a^ | ＞0.05 |
| Shehabi et al^28^ (2012) | 171/80 | Midazolam/  Diazepam | - | - | Unclear | - |
| van den Boogaard et al^29^ (2012) | 249/ 1017 | - | - | - | Unclear | - |
| Shehabi et al^19^ (2013) | 16/21 | Midazolam | 0.3 vs 0.06 ^5^ | 0.036 | 18.6 vs 20.2 ^a^ | ＞0.05 |
| Hager et al^30^ (2013) | 120/82 | Benzodiazepine | 70 vs 22 ^6^ | ＜0.001 | 29 vs 29 ^a^ | 0.703 |
| Shehabi et al^31^ (2013) | 30/30 | Midazolam | 100 vs 4.6 ^6^ | ＜0.05 | 18 vs 20 ^a^ | ＞0.05 |
| Nassar et al^32^ (2014) | 30/30 | Midazolam | 45 vs 0 ^3^ | ＜0.001 | 18 vs 22 ^a^ | ＞0.05 |
| Dale et al^33^ (2014) | 703/780 | Lorazepam | 49.2 vs 17.2 ^3^ | ＜0.01 | 43.8 vs 42.2 ^b^ | 0.06 |
| Balzer et al^34^ (2015) | 513/ 1371 | Midazolam | 99 vs 26 ^6^ | ＜0.001 | 25 vs 20 ^a^ | ＜0.001 |
| Skrupky et al^35^ (2015) | 97/102 | Midazolam | 0.03 vs 0.02 ^4^ | 0.0124 | 21 vs 19 ^a^ | 0.800 |
| Stephens et al^36^ (2017) | 231/132 | - | - | - | Unclear | - |
| Kawazoe et al^37^ (2017) | 101/100 | Midazolam | 22 vs 0 ^2^ | ＜0.05 | 22 vs 23 ^a^ | ＞0.05 |
| Kaplan et al^38^ (2018) | 66/66 | Midazolam | 10.4 vs 2 ^2^ | ＜0.001 | 55.2 vs 48.6 ^b^ | 0.03 |
| De Jonghe et al^39^ (2018) | 590/584 | Midazolam | 263 vs 218 ^3^ | 0.03 | 54.4 vs 53.6 ^b^ | ＞0.05 |

D/L: Deeper/Lighter gourp, BDZ: benzodiazepine (Different BDZ means there was a significant difference in BDZ dosage between two groups), ^1^: mg/h, ^2^: mg/d, ^3^: mg(cumulative dose) ^4^: mg/kg/h, ^5^: mg/kg(cumulative dose), ^6^: %(% of patient midazolam was given), -: means without BZD or data unavailable. ^a^: APACHEⅡ(Acute Physiology and Chronic Health EvaluationⅡ), ^b^: SAPSⅡ (Simplified Acute Physiology ScoreⅡ), ^C^: SOFA (Sequential Organ Failure Assessment)
