# Supplementary material for: Different depths of sedation versus risk of delirium in adult mechanically ventilated patients: A systematic review and meta-analysis
Source: PLoS One. 2020 Jul 16;15(7):e0236014. doi: 10.1371/journal.pone.0236014 (PMC7365415; doi:10.1371/journal.pone.0236014)
Supplement: S2 Table — (DOC) [file pone.0236014.s005.doc]

| Cochrane Bias Assessment of Randomized Studies | | | | | | | |
| --- | --- | --- | --- | --- | --- | --- | --- |
| Author,  Year | Selection  Bias Risk | Allocation  Bias Risk | Performance  bias Risk | Detection  Bias Risk | Attrition  Bias Risk | Reporting  Bias Risk | Other  Bias Risk |
| Pandharipande, 2007 | Low  Patients were randomized using conputer-generated, permuted block randomization | Low  Only pharmacist were known to study drug assignment | Low  Patients and study personnel were blinded to study drug allocation | Low  Participant assessment was blinded | Low  No important outcome data missing | Low  All of the pre-specified outcomes of interest in the review have been reported. | Low  Study appears to be free of other sources of bias |
| Samuelson, 2008 | Low  The Randomization sequence was generated from a statistical table of random numbers | Low  Concealed envelopes used | Low  Patients and study personnel were blinded to study drug allocation | Low  Participant assessment was blinded | Low  No important outcome data missing | Low  All of the pre-specified outcomes of interest in the review have been reported | Low  Study appears to be free of other sources of bias |
| Girad, 2008 | Low  Patients were randomized using conputer-generated, permuted block randomization | Low  Concealed envelopes used | High  Study personnel unblinded to treatment group allocation | High  Participant assessment was unblinded | Low  No important outcome data missing | Low  All of the pre-specified outcomes of interest in the review have been reported | Low  Study appears to be free of other sources of bias |
| Treggiari, 2009 | Low  Patients were randomized using computer-generated sequence of random numbers | Low  Method of allocation not described, however study describes use of concealed envelopes | High  the study was single blind | High  Outcome assessment was unblinded | Low  No important outcome data missing | Low  All of the pre-specified outcomes of interest in the review have been reported | Low  Study appears to be free of other sources of bias |
| Strøm, 2010 | Unclear  Method of randomization not described | Low  Concealed envelopes used | High  Participants and investigators both unblinded to treatment group allocation | High  Participant assessment was unblinded | Low  No important outcome data missing | Low  All of the pre-specified outcomes of interest in the review have been reported | Low  Study appears to be free of other sources of bias |
| Shehabi, 2013  (Australia) | Low  Patients randomly allocated using block randomization | Low  Concealed envelopes used | High  Participants and investigators both unblinded to treatment group allocation | High  Participant assessment was unblinded | Low  No important outcome data missing | Low  All of the pre-specified outcomes of interest in the review have been reported | Low  Study appears to be free of other sources of bias |
| Shehabi, 2013  (Malaysian) | Low  Method of randomization not described, however study protocol describes block randomization | Low  Method of allocation not described, however study protocol describes use of concealed envelopes | High  Participants and investigators both unblinded to treatment group allocation | High Participant assessment was unblinded | Low  No important outcome data missing | Low  Study protocol is available and all of the pre-specified outcomes of interest in the review have been reported. | Low  Study appears to be free of other sources of bias |
| Nassar, 2014 | Unclear  Method of randomization not described | Low  Concealed envelopes used | High  Participants and investigators both unblinded to treatment group allocation | High Participant assessment was unblinded | Low  No important outcome data missing | Low  Study protocol is available and all of the pre-specified outcomes of interest in the review have been reported. | Low  Study appears to be free of other sources of bias |
| Kawazoe, 2017 | Low  Patients randomly allocated using block randomization | High  Allocation of open labels | High  Participants and investigators both unblinded to treatment group allocation | High Participant assessment was unblinded | Low  No important outcome data missing | Low  Study protocol is available and all of the pre-specified outcomes of interest in the review have been reported. | Low  Study appears to be free of other sources of bias |
| De Jonghe, 2018 | Low  Patients randomly allocated using block randomization | Unclear  Method of allocation not described | High  Participants and investigators both unblinded to treatment group allocation | High Participant assessment was unblinded | Low  No important outcome data missing | Low  Study protocol is available and all of the pre-specified outcomes of interest in the review have been reported. | Low  Study appears to be free of other sources of bias |
