# Supplementary material for: Different depths of sedation versus risk of delirium in adult mechanically ventilated patients: A systematic review and meta-analysis
Source: PLoS One. 2020 Jul 16;15(7):e0236014. doi: 10.1371/journal.pone.0236014 (PMC7365415; doi:10.1371/journal.pone.0236014)
Supplement: S1 File — (DOC) [file pone.0236014.s006.doc]

CENTRAL search strategy

#1 MeSH descriptor: [Conscious Sedation] explode all trees

#2 MeSH descriptor: [Deep Sedation] explode all trees

#3 (sedation): ti,ab,kw OR (deep sedation): ti,ab,kw OR (heavy sedation): ti,ab,kw OR (over sedation): ti,ab,kw OR (over-sedation): ti,ab,kw OR (conscious sedation): ti,ab,kw OR (moderate sedation): ti,ab,kw OR (under-sedation): ti,ab,kw OR (poor sedation): ti,ab,kw

#4 #1 OR #2 OR #3

#5 MeSH descriptor: [Delirium] explode all trees

#6 MeSH descriptor: [Dementia] explode all trees

#7 (delirium) OR (dementia)

#8 #5 OR #6 OR #7

#9 MeSH descriptor: [Randomized Controlled Trial] explode all trees

#10 MeSH descriptor: [Controlled Clinical Trial] explode all trees

#11 MeSH descriptor: [Cohort Study] explode all trees

#12 MeSH descriptor: [Controlled before-after Study] explode all trees

#13 (randomized controlled trial): pt OR (randomized clinical trial): pt OR (cohort study): pt OR (controlled before-after study): pt

#14 #9 OR #10 OR #11 OR #12 OR #13

#15 MeSH descriptor: [Respiration, Artificial] explode all trees

#16 (mechanical ventilation): ti,ab,kw OR (respiration artificial): ti,ab,kw

#17 #15 OR #16

#18 #4 AND #8 AND #14 AND #17

Pubmed search strategy

#1 (((((conscious sedation[MeSH Terms]) OR deep sedation[MeSH Terms]) OR moderate sedation[MeSH Terms]))) OR ((((((((((sedation[Title/Abstract]) OR heavy sedation[Title/Abstract]) OR over sedation[Title/Abstract]) OR deep sedation[Title/Abstract]) OR conscious sedation[Title/Abstract]) OR light sedation[Title/Abstract]) OR moderate sedation[Title/Abstract]) OR under sedation[Title/Abstract]) OR poor sedation[Title/Abstract]))

#2 ((((delirium[MeSH Terms]) OR dementia[MeSH Terms]))) OR (((delirium) OR dementia))

#3 ((((cohort study[MeSH Terms]) OR controlled before-after study[MeSH Terms]) OR ((randomized controlled trial[MeSH Terms]) OR controlled clinical trial[MeSH Terms]))) OR ((((randomized controlled trial[Publication Type]) OR controlled clinical trial[Publication Type]) OR cohort study[Publication Type]) OR controlled before-after study[Publication Type])

#4 (((mechanical ventilation[MeSH Terms]) OR respiration artificial[MeSH Terms]) OR respiration artificial[Title/Abstract]) OR mechanical ventilation[Title/Abstract]

#5 (((animal[MeSH Terms]) AND humans[MeSH Terms])) OR animal[MeSH Terms]

#6 #1 AND #2 AND #3 AND #4 NOT #5

Embase search strategy

#1 'sedation':ti,ab,kw OR 'conscious sedation':ti,ab,kw OR 'deep sedation':ti,ab,kw OR 'heave sedation':ti,ab,kw OR 'over sedation':ti,ab,kw OR 'light sedation':ti,ab,kw OR 'moderate sedation':ti,ab,kw OR 'under sedation':ti,ab,kw OR 'poor sedation':ti,ab,kw

#2 delirium OR dementia

#3 'randomized controlled trial':pt OR 'randomized clinical trial':pt OR 'cohort study':pt OR 'controlled before-after study':pt

#4 'mechanical ventilation':ti,ab,kw OR 'respiration artificial':ti,ab,kw

#5 animals OR (humans and animals)

#6 #1 AND #2 AND #3 AND #4 NOT #5

CINAHL search strategy

S1 MW sedation OR MW deep sedation OR MW conscious sedation OR AB heavy sedation OR AB over sedation OR AB light sedation OR AB moderate sedation OR AB under sedation OR AB poor sedation

S2 TX delirium OR TX dementia

S3 MM randomized controlled trials OR MM randomized clinical trial OR MM controlled trials OR MM cohort study

MM controlled before-after study

S4 MH mechanical ventilation OR MH respiration, artificial

S4 S1 AND S2 AND S3 AND S4
