# Supplementary material for: Different depths of sedation versus risk of delirium in adult mechanically ventilated patients: A systematic review and meta-analysis
Source: PLoS One. 2020 Jul 16;15(7):e0236014. doi: 10.1371/journal.pone.0236014 (PMC7365415; doi:10.1371/journal.pone.0236014)
Supplement: S2 File — (DOCX) [file pone.0236014.s007.docx]

The table of GRADE

| Outcome | Relative effect(OR) | No of Participants | Quality of the evidence |
| --- | --- | --- | --- |
| incidence of delirium | 1.00(0.64 to 1.58) | 8001 | ➕➕➕➖（moderate) |
| incidence of agitation-related adverse events | 0.61(0.45 to 0.84) | 2282 | ➕➕➕➕（high) |
| mortality | 1.82(1.23 to 2.69) | 7804 | ➕➕➕➖（moderate) |
