# Supplementary material for: Different depths of sedation versus risk of delirium in adult mechanically ventilated patients: A systematic review and meta-analysis
Source: PLoS One. 2020 Jul 16;15(7):e0236014. doi: 10.1371/journal.pone.0236014 (PMC7365415; doi:10.1371/journal.pone.0236014)
Supplement: S3 File — (DOCX) [file pone.0236014.s008.docx]

**STEP 1: 130 duplicate articles removed**

Pubmed and Embase and CENTRAL and CIHAHL(42 items)

1. Shehabi Y, Howe BD, Bellomo R, et al. Early Sedation with Dexmedetomidine in Critically Ill Patients.[J]. N Engl J Med, 2019, 380(26):2506-2517.
2. Nedergaard HK, Jensen HI, Stylsvig M, et al. Non-sedation versus sedation with a daily wake-up trial in critically ill patients receiving mechanical ventilation- effects on long-term cognitive function: study protocol for a randomized controlled trial, a substudy of the NONSEDA trial.[J]. Trials, 2016, 17(1):269.
3. Khan SH, Wang S, Harrawood A, et al. Decreasing Delirium through Music (DDM) in critically ill, mechanically ventilated patients in the intensive care unit: study protocol for a pilot randomized controlled trial.[J]. Trials, 2017, 18(1):574.
4. Kawazoe Y, Miyamoto K, Morimoto T, et al. Effect of Dexmedetomidine on Mortality and Ventilator-Free Days in Patients Requiring Mechanical Ventilation With Sepsis: a Randomized Clinical Trial.[J]. JAMA, 2017, 317(13):1321-1328.
5. Jerath A, Ferguson ND, Steel A, et al. The use of volatile anesthetic agents for long-term critical care sedation (VALTS): study protocol for a pilot randomized controlled trial.[J]. Trials, 2015, 16(1):560.
6. Reade MC, Eastwood GM, Bellomo R, et al. Effect of Dexmedetomidine Added to Standard Care on Ventilator-Free Time in Patients With Agitated Delirium: A Randomized Clinical Trial.[J]. JAMA, 2016, 315(14):1460-1468.
7. Al-Qadheeb NS, Skrobik Y, Schumaker G, et al. Preventing ICU Subsyndromal Delirium Conversion to Delirium With Low-Dose IV Haloperidol: a Double-Blind, Placebo-Controlled Pilot Study.[J]. Crit Care Med, 2016, 44(3):583-591.
8. Mehta S, Burry L, Cook D, et al. Daily sedation interruption in mechanically ventilated critically ill patients cared for with a sedation protocol: a randomized controlled trial.[J]. JAMA, 2012, 308(19):1985-1992.
9. Mansouri P, Javadpour S, Zand F, et al. [Implementation of a protocol for integrated management of pain, agitation, and delirium can improve clinical outcomes in the intensive care unit: a randomized clinical trial](https://www.cochranelibrary.com/central/doi/10.1002/central/CN-00883391/full).[J]. J Crit Care, 2013, 28(6):918-922.
10. MacLaren R, Preslaski CR, Mueller SW, et al. A randomized, double-blind pilot study of dexmedetomidine versus midazolam for intensive care unit sedation: patient recall of their experiences and short-term psychological outcomes.[J]. J Intensive Care Med, 2015, 30(3):167-175.
11. Riker RR, Shehabi Y, Bokesch PM, et al. Dexmedetomidine vs midazolam for sedation of critically ill patients: a randomized trial.[J]. JAMA, 2009, 301(5):489-499.
12. Toft P, Olsen HT, Jørgensen HK, et al. Non-sedation versus sedation with a daily wake-up trial in critically ill patients receiving mechanical ventilation (NONSEDA Trial): study protocol for a randomised controlled trial.[J]. Trials, 2014, 15(1):499.
13. Pandharipande PP, Sanders RD, Girard TD, et al. Effect of dexmedetomidine versus lorazepam on outcome in patients with sepsis: an a priori-designed analysis of the MENDS randomized controlled trial.[J]. Crit Care, 2010, 14(2):R38.
14. Reade MC, O'Sullivan K, Bates S, et al. Dexmedetomidine vs. haloperidol in delirious, agitated, intubated patients: a randomised open-label trial.[J]. Crit Care. 2009, 13(3):R75.

Pubmed and Embase and CENTRAL(44 items)

1. Wit MD, Gennings C, Jenvey WI, et al. Randomized trial comparing daily interruption of sedation and nursing-implemented sedation algorithm in medical intensive care unit patients.[J]. Critical Care, 2008, 12(3):R70.
2. Liu D, Lyu J, Zhao H, et al. The influence of analgesic-based sedation protocols on delirium and outcomes in critically ill patients: a randomized controlled trial.[J]. PLoS One, 2017, 12(9):e0184310.
3. Mehta S, Cook D, Devlin JW, et al. Prevalence, Risk Factors, and Outcomes of Delirium in Mechanically Ventilated Adults.[J]. Crit Care Med, 2015, 43(3):557-566.
4. Pandharipande PP, Pun BT, Herr DL, et al. Effect of sedation with dexmedetomidine vs lorazepam on acute brain dysfunction in mechanically ventilated patients: the MENDS randomized controlled trial.[J]. JAMA, 2007, 298(22):2644-2653.
5. Elgebaly AS, Sabry M. Sedation effects by dexmedetomidine versus propofol in decreasing duration of mechanical ventilation after open heart surgery.[J]. Ann Card Anaesth, 2018, 21(3):235-242.
6. Corbett SM, Rebuck JA, Greene CM, et al. Dexmedetomidine does not improve patient satisfaction when compared with propofol during mechanical ventilation.[J]. Crit Care Med, 2005, 33(5):940-945.
7. Muellejans B, Matthey T, Scholpp J, et al. Sedation in the intensive care unit with remifentanil/propofol versus midazolam/fentanyl: a randomised, open-label, pharmacoeconomic trial.[J]. Crit Care, 2006, 10(3):R91.
8. Mistraletti G, Umbrello M, Salini S, et al. Enteral versus intravenous approach for the sedation of critically ill patients: a randomized and controlled trial.[J]. Crit Care, 2019, 23(1):3.
9. Lu X, Li J, Li T, et al. Clinical study of midazolam sequential with dexmedetomidine for agitated patients undergoing weaning to implement light sedation in intensive care unit.[J]. Chin J Traumatol, 2016, 19(2):94-96.
10. Liu X, Zhang K, Wang W, et al. Dexmedetomidine Versus Propofol Sedation Improves Sublingual Microcirculation After Cardiac Surgery: a Randomized Controlled Trial.[J]. J Cardiothorac Vasc Anesth, 2016, 30(6):1509-1515.
11. Girard TD, Exline MC, Carson SS, etl al. Haloperidol and Ziprasidone for Treatment of Delirium in Critical Illness.[J]. N Engl J Med, 2018, 379(26):2506-2516.
12. Perbet S, Verdonk F, Godet T, et al. Low doses of ketamine reduce delirium but not opiate consumption in mechanically ventilated and sedated ICU patients: A randomised double-blind control trial.[J]. Anaesth Crit Care Pain Med, 2018, 37(6):589-595.
13. Yu HJ, Xu XQ, Xu SA, et al. Analgesic and Sedative Effect of Acupuncture Combined with Medicine on Patients Undergiong Cardiac Surgery.[J]. Zhongguo zhong xi yi jie he za zhi, 2016, 36(3):289-293.
14. Song R, Li J, Dong C, et al. A study of using dexmedetomidine in ventilator bundle treatment in an ICU.[J]. Zhonghua wei zhong bing ji jiu yi xue, 2015, 27(10):836-840.
15. Lyu J, Liu D, An Y, et al. The influence of the sedation based on remifentanil analgesia on the occurrence of delirium in critically ill patients.[J]. Zhonghua wei zhong bing ji jiu yi xue, 2015, 27(10):845-849.
16. Huang F, Wang J, Yang X, et al. Sedative effects of dexmedetomidine in post-operative elder patients on mechanical ventilation.[J]. Zhonghua yi xue za zhi, 2014, 94(41):3211-3215.
17. Eremenko AA, Chemova EV. Comparison of dexmedetomidine and propofol for short-term sedation in early postoperative period after cardiac surgery.[J]. Anesteziol Reanimatol, 2014, (2):37-41.
18. Shehabi Y, Bellomo R, Reade MC, et al. Early goal-directed sedation versus standard sedation in mechanically ventilated critically ill patients: a pilot study*.[J]. Crit Care Med, 2013, 41(8):1983-1991.
19. Aydogan MS, Korkmaz MG, Ozgul U, et al. Pain, fentanyl consumption, and delirium in adolescents after scoliosis surgery: dexmedetomidine vs midazolam.[J]. Paediatr Anaesth, 2013, 23(5):446-452.
20. Eremenko AA, Chernova EV. Dexmedetomidine use for intravenous sedation and delirium treatment during early postoperative period in cardio-surgical patients.[J]. Anesteziol Reanimatol, 2013, 5:4-8.
21. Pohlman MC, Schweickert WD, Pohlman AS, et al. Feasibility of physical and occupational therapy beginning from initiation of mechanical ventilation.[J]. Crit Care Med, 2010, 38(11):2089-2094.
22. Schweichert WD, Pohlman MC, Pohlman AS, et al. Early physical and occupational therapy in mechanically ventilated, critically ill patients: a randomised controlled trial.[J]. Lancet, 2009, 373(9678):1874-1882.

Pubmed and CINAHL (27 items)

1. Brophy A, Cardinale M, Andrews L, et al. Prospective Observational Evaluation of Sedation and Pain Management Guideline Adherence Across New Jersey Intensive Care Units.[J]. J Pharm Pract, 2019, 32(5):529-533.
2. Roberts M, Johnson S, Weyant RA, et al. The Experience of Acute Mechanical Ventilation From the Patient's Perspective.[J]. Dimens Crit Care Nurs, 2019, 38(4):201-212.
3. Trogrlic Z, van der Jagt M, Lingsma H, et al. Improved Guideline Adherence and Reduced Brain Dysfunction After a Multicenter Multifaceted Implementation of ICU Delirium Guidelines in 3,930 Patients.[J]. Crit Care Med, 2019, 47(3):419-427.
4. Dale CR, Kannas DA, Fan VS, et al. Improved analgesia, sedation, and delirium protocol associated with decreased duration of delirium and mechanical ventilation.[J]. Ann Am Thorac Soc, 2014, 11(3):367-374.
5. Dale CR, Bryson CL, Fan VS, et al. A greater analgesia, sedation, delirium order set quality score is associated with a decreased duration of mechanical ventilation in cardiovascular surgery patients.[J]. Crit Care Med, 2013, 41(11):2610-2617.
6. Kamdar BB, Combs MP, Colantuoni E, et al. The association of sleep quality, delirium, and sedation status with daily participation in physical therapy in the ICU.[J]. 2016, 19:261.
7. Brenda TP, Michele CB, Mary A, et al. Caring for Critically Ill Patients with the ABCDEF Bundle: Results of the ICU Liberation Collaborative in Over 15,000 Adults.[J]. Crit Care Med, 2019, 47(1):3-14.
8. Khan RM, Al-Juaid M, Al-Mutairi H, et al. Implementing the comprehensive unit-based safety program model to improve the management of mechanically ventilated patients in Saudi Arabia.[J]. Am J Infect Control, 2019, 47(1):51-58.
9. Shehabi Y, Bellomo R, Kadiman S, et al. Sedation Intensity in the First 48 Hours of Mechanical Ventilation and 180-Day Mortality: A Multinational Prospective Longitudinal Cohort Study.[J]. Crit Care Med, 2018. 46(6):850-859.
10. Seymour CW, Pandharipande PP, Koestner T, et al. Diurnal sedative changes during intensive care: impact on liberation from mechanical ventilation and delirium.[J]. Crit Care Med, 2012, 40(10):2788-2796.
11. Brummel NE, Jackson JC, Pandharipande PP, et al. Delirium in the ICU and subsequent long-term disability among survivors of mechanical ventilation.[J]. Crit Care Med, 2014, 42(2):369-377.
12. Hsieh SJ, Soto GJ, Hope AA, et al. The association between acute respiratory distress syndrome, delirium, and in-hospital mortality in intensive care unit patients.[J]. Am J Respir Crit Care Med, 2015, 191(1):71-78.
13. Schickli MA, Eberwein KA, Short MR, et al. Pharmacy-Driven Dexmedetomidine Stewardship and Appropriate Use Guidelines in a Community Hospital Setting.[J]. Ann Pharmacother, 2017, 51(1):27-32.
14. McAndrew NS, Leske J, Guttormson J, et al. Quiet time for mechanically ventilated patients in the medical intensive care unit.[J]. Intensive Crit Care Nurs, 2016, 35:22-27.
15. Robinson BR, Mueller EW, Henson K, et al. An analgesia-delirium-sedation protocol for critically ill trauma patients reduces ventilator days and hospital length of stay.[J]. J Trauma, 2008, 65(3):517-526.
16. Castillo MI, Cooke M, Macfarlane B, et al. Factors associated with anxiety in critically ill patients: A prospective observational cohort study.[J]. Int J Nurs Stud, 2016, 60:225-233.
17. Liu V, Herbert D, Foss-Durant A, et al. Evaluation Following Staggered Implementation of the "Rethinking Critical Care" ICU Care Bundle in a Multicenter Community Setting.[J]. Crit Care Med, 2016, 44(3):460-467.
18. Shehabi Y, Riker RR, Bokesch PM, et al. Delirium duration and mortality in lightly sedated, mechanically ventilated intensive care patients.[J]. Crit Care Med, 2010, 38(12):2311-2318.
19. Gold JA, Rimal B, Nolan A, et al. A strategy of escalating doses of benzodiazepines and phenobarbital administration reduces the need for mechanical ventilation in delirium tremens.[J]. Crit Care Med, 2007, 35(3):724-730.
20. Slomka J, Hoffman-Hogg L, Mion LC, et al. Influence of clinicians' values and perceptions on use of clinical practice guidelines for sedation and neuromuscular blockade in patients receiving mechanical ventilation.[J]. Am J Crit Care, 2000, 9(6):412-418.
21. Tomasi CD, Grandi C, Salluh J, et al. Comparison of CAM-ICU and ICDSC for the detection of delirium in critically ill patients focusing on relevant clinical outcomes.[J]. J Crit Care, 2012, 27(2):212-217.
22. Pandharipande P, Cotton BA, Shintani A, et al. Prevalence and risk factors for development of delirium in surgical and trauma intensive care unit patients.[J]. J Trauma, 2008, 65(1):34-41.
23. Ely EW, Shintani A, Truman B, et al. Delirium as a predictor of mortality in mechanically ventilated patients in the intensive care unit.[J]. JAMA, 2004, 291(14):1753-1762
24. Mendez-Tellez PA, Dinglas VD, Colantuoni E, et al. Factors associated with timing of initiation of physical therapy in patients with acute lung injury.[J]. J Crit Care, 2013, 28(6):980-984.
25. Fan E, Shahid S, Kondreddi VP, et al. Informed consent in the critically ill: a two-step approach incorporating delirium screening.[J]. Crit Care Med, 2008, 36(1):94-99.
26. Balas MC, Happ MB, Yang W, et al. Outcomes Associated With Delirium in Older Patients in Surgical ICUs.[J].Chest, 2009, 135(1):18-25.
27. Chen K, Lu Z, Xin YC, et al. Alpha-2 agonists for long-term sedation during mechanical ventilation in critically ill patients.[J]. Cochrane Database Syst Rev, 2015, 1(1):CD010269.

Pubmed and CENTRAL（13 items）

1. Nilsen ML, Sereika S, Happ MB. Nurse and patient characteristics associated with duration of nurse talk during patient encounters in ICU.[J]. Heart Lung, 2013, 42(1):5-12.
2. Dessap AM, Roche-Campo F, Launay JM, et al. Delirium and Circadian Rhythm of Melatonin During Weaning From Mechanical Ventilation: an Ancillary Study of a Weaning Trial.[J]. Chest, 2015,148(5):1231-1241.
3. Girard TD, Pandharipande PP, Carson SS, et al. Feasibility, efficacy, and safety of antipsychotics for intensive care unit delirium: the MIND randomized, placebo-controlled trial.[J]. Crit Care Med, 2010, 38(2):428-437.
4. Strøm T, Martinussen T, Toft P. A protocol of no sedation for critically ill patients receiving mechanical ventilation: a randomized trial.[J]. Lancet, 2010, 375(9713):475-480.
5. Neubert A, Baarslag MA, Dijk MV, et al. The CLOSED trial; CLOnidine compared with midazolam for SEDation of paediatric patients in the intensive care unit: study protocol for a multicentre randomised controlled trial.[J]. BJM open,2017, 7(6):e016031.
6. Wan LJ, Huang QQ, Yue JX, et al. Comparison of sedative effect of dexmedetomidine and midazolam for post-operative patients undergoing mechanical ventilation in surgical intensive care unit.[J]. Zhonghua wei zhong bing ji jiu yi xue, 2011, 23(9):543-546.(中文)
7. Nadler JW, Evans JL, Fang E, et al. A randomised trial of peri-operative positive airway pressure for postoperative delirium in patients at risk for obstructive sleep apnoea after regional anaesthesia with sedation or general anaesthesia for joint arthroplasty.[J]. Anaesthesia, 2017, 72(6):729-736.
8. Stollings JL, Thompson JL, Ferrell BA, et al. Sedative Plasma Concentrations and Delirium Risk in Critical Illness.[J]. Ann Pharmacother, 2018, 52(6):513-521.
9. Huber FT, Bartels H, Siewert JR. Treatment of postoperative alcohol withdrawal syndrome after esophageal resection.[J]. Langenbecks Arch Chir Suppl II verh Dtsch Ges Chir, 1990, 4:1141-1143.
10. Kim MS, Moon BE, Kim H, et al. Comparison of propofol and fentanyl administered at the end of anesthesia for prevention of emergence agitation after sevoflurane anaesthesia in children.[J]. Br J Anaesth, 2013,110(2):274-280.
11. Soja SL, Pandharipande PP, Fleming SB, et al. Implementation, reliability testing, and compliance monitoring of the Confusion Assessment Method for the Intensive Care Unit in trauma patients.[J]. Intensive Care Med, 2008,34(7):1263-1268.
12. Burry L, Cook D, Herridge M, et al. Recall of ICU Stay in Patients Managed With a Sedation Protocol or a Sedation Protocol With Daily Interruption.[J]. Crit Care Med, 2015, 43(10):2180-2190.
13. Vourc’h M, Feuillet F, Mahe PJ, et al. Baclofen to prevent agitation in alcohol-addicted patients in the ICU: study protocol for a randomised controlled trial.[J]. Trials, 2016, 17(1):415.

Pubmed and Embase (4 items)

1. Kiski D, Malec E, Schmidt C. [Use of dexmedetomidine in pediatric cardiac anesthesia](https://www-embase-com.ezproxy.lb.polyu.edu.hk/a/#/search/results?subaction=viewrecord&rid=11&page=1&id=L631345226).[J]. Curr Opin Anaesthesiol, 2019, 32(3):334-342.
2. Louis C, Godet T, Chanques G, et al. Effects of dexmedetomidine on delirium duration of non-intubated ICU patients (4D trial): study protocol for a randomized trial.[J]. Trials, 2018, 19(1):307.
3. Wilson J, Carlson R, Duggan MC, et al. Delirium and Catatonia in Critically Ill Patients: The Delirium and Catatonia Prospective Cohort Investigation.[J]. Crit Care Med, 2017, 45(11):1837-1844
4. Burry LD, Williamson DR, Perreault MM, et al. [Analgesic, sedative, antipsychotic, and neuromuscular blocker use in Canadian intensive care units: A prospective, multicentre, observational study](https://www-embase-com.ezproxy.lb.polyu.edu.hk/a/#/search/results?subaction=viewrecord&rid=88&page=1&id=L53172725).[J]. Can J Anesth, 2014, 61(7):619-630.

**STEP 2: 158 records exclude by tittle and abstract**

Study types failed to meet the inclusion criteria

1. Guttormson JL, Chlan L, Tracy MF, et al. Nurses' Attitudes and Practices Related to Sedation: A National Survey.[J]. Am J Crit Care, 2019, 28(4):255-263.
2. Pavone KJ, Cacchione PZ, Polomano RC, et al. Evaluating the use of dexmedetomidine for the reduction of delirium: An integrative review.[J]. Heart Lung, 2018, 47(6):591-601.
3. Stephens RJ, Dettmer MR, Roberts BW, et al. Practice Patterns and Outcomes Associated With Early Sedation Depth in Mechanically Ventilated Patients: A Systematic Review and Meta-Analysis.[J]. Crit Care Med, 2018, 46(3):471-479.
4. Rock LF. Sedation and Its Association With Posttraumatic Stress Disorder After Intensive Care.[J]. Crit Care Nurse, 2014, 34(1):30-37.
5. Reardon DP, Anger KE, Adams CD, et al. Role of dexmedetomidine in adults in the intensive care unit: An update.[J]. Am J Health Syst Pharm, 2013, 70(9):767-777.
6. Minhas MA, Velasquez AG, Kaul A, et al. Effect of Protocolized Sedation on Clinical Outcomes in Mechanically Ventilated Intensive Care Unit Patients: A Systematic Review and Meta-analysis of Randomized Controlled Trials.[J]. Mayo Clin Proc, 2015, 90(5):613-623.
7. Hughes CG, Girard TD, Pandharipande PP. Daily sedation interruption versus targeted light sedation strategies in ICU patients.[J]. Crit Care Med, 2013, 41(9 Suppl 1):S39-45.
8. Roberts DJ, Haroon BH, Babar, Richard I. Sedation for Critically Ill or Injured Adults in the Intensive Care Unit: A Shifting Paradigm.[J]. Drugs, 2012, 72(14):1881-1916.
9. Slomka J, Hoffman-Hogg L, Mion LC, et al. Influence of clinicians' values and perceptions on use of clinical practice guidelines for sedation and neuromuscular blockade in patients receiving mechanical ventilation.[J]. J Crit Care, 2000, 9(6):412-418.
10. Van Rompaey B, Elseviers MM, Schuurmans MJ, et al. Risk factors for delirium in intensive care patients: a prospective cohort study.[J]. Crit Care, 2009, 13(3):R77.
11. Riker RR, Fraser GL. Altering intensive care sedation paradigms to improve patient outcomes.[J]. Crit Care Clin, 2009, 25(3):527-538.
12. [DeBiasi](https://pubmed.ncbi.nlm.nih.gov/?term=DeBiasi+EM&cauthor_id=26595050) EM, [Akgün](https://pubmed.ncbi.nlm.nih.gov/?term=Akg%C3%BCn+KM&cauthor_id=26595050) KM, [Pisani](https://pubmed.ncbi.nlm.nih.gov/?term=Pisani+M&cauthor_id=26595050) M. Awake or Sedated: Trends in the Evaluation and Management of Agitation in the Intensive Care Unit.[J].Semin Respir Crit Care Med, 2015, 36(6):899-913.
13. Kress JP. The complex interplay between delirium, sepsis and sedation.[J]. Crit Care, 2010, 14(3):164.
14. Rathier MO, Baker WL. A review of recent clinical trials and guidelines on the prevention and management of delirium in hospitalized older patients.[J]. Hosp Pract (1995), 2011, 39(4):96-106.
15. Luetz A, Goldmann A, Weber-Carstens S, et al. Weaning from mechanical ventilation and sedation.[J]. Curr Opin Anaesthesiol, 2012, 25(2):164-169.
16. McGrane S, Pandharipande PP. Sedation in the intensive care unit.[J]. Minerva Anestesiol, 2012, 78(3):369-380.
17. Lin YY, He B, Chen J, et al. Can dexmedetomidine be a safe and efficacious sedative agent in post-cardiac surgery patients? a meta-analysis.[J]. Crit Care, 2012, 16(5):R169.
18. Fraser GL, Devlin JW, Worby CP, et al. Benzodiazepine versus nonbenzodiazepine-based sedation for mechanically ventilated, critically ill adults: a systematic review and meta-analysis of randomized trials.[J]. Crit Care Med, 2013, 41(9 Suppl 1):S30-38.
19. Xia ZQ, Chen SQ, Yao X, et al. Clinical benefits of dexmedetomidine versus propofol in adult intensive care unit patients: a meta-analysis of randomized clinical trials.[J]. J Surg Res, 2013, 185(2):833-843.
20. Burry L, Rose L, McCullagh IJ, et al. Daily sedation interruption versus no daily sedation interruption for critically ill adult patients requiring invasive mechanical ventilation.[J]. Cochrane Database Syst Rev, 2014, 2014(7):CD009176.
21. Chen K, Lu Z, Xin YC, et al. Alpha-2 agonists for long-term sedation during mechanical ventilation in critically ill patients.[J]. Cochrane Database Syst Rev, 2015, 1(1):CD010269.
22. Ntoumenopoulos G. Rehabilitation during mechanical ventilation: Review of the recent literature.[J]. Intensive Crit Care Nurs, 2015, 31(3):125-132.
23. Minhas MA, Velasquez AG, Kaul A, et al. Effect of Protocolized Sedation on Clinical Outcomes in Mechanically Ventilated Intensive Care Unit Patients: A Systematic Review and Meta-analysis of Randomized Controlled Trials.[J]. Mayo Clin Proc, 2015, 90(5):613-623.
24. Constantin JM, Momon A, Mantz J, et al. Efficacy and safety of sedation with dexmedetomidine in critical care patients: a meta-analysis of randomized controlled trials.[J]. Anaesth Crit Care Pain Med, 2016, 35(1):7-15.
25. Nassar AP Junior, Park M. Sedation protocols versus daily sedation interruption: a systematic review and meta-analysis.[J]. Rev Bras Ter Intensiva, 2016, 28(4):444-451.
26. Pandharipande PP, Ely EW, Arora RC, et al. The intensive care delirium research agenda: a multinational, interprofessional perspective.[J]. Intensive Care Med, 2017, 43(9):1329-1339.
27. Koopmans M, Vermei L, van Wieren A, et al. Improvement in functional abilities at ICU discharge is feasible without prolongation of the length of stay ICU.[J]. Intensive Care Med, 2017, 43(2):273-274.
28. Herling SF, Greve IE, Vasilevskis EE, et al. Interventions for preventing intensive care unit delirium in adults.[J]. Cochrane Database Syst Rev, 2018, 11(11):CD009783.
29. Shetty RM, Bellini A, Wijayatilake DS, et al. BIS monitoring versus clinical assessment for sedation in mechanically ventilated adults in the intensive care unit and its impact on clinical outcomes and resource utilization.[J]. Cochrane Database Syst Rev, 2018, 2(2):CD011240.
30. Fernandez-Gonzalo S, Turon M, De Haro C, et al. Do sedation and analgesia contribute to long-term cognitive dysfunction in critical care survivors?.[J]. Med Intensiva, 2018, 42(2):114-128.
31. Rengel KF, Hayhurst CJ, Pandharipande PP, et al. Long-term Cognitive and Functional Impairments After Critical Illness.[J]. Anesth Analg, 2019, 128(4):772-780.
32. Dirkes SM, Kozlowski C. Early Mobility in the Intensive Care Unit: Evidence, Barriers, and Future Directions.[J]. Crit Care Nurse, 2019, 39(3):33-42.
33. Thrane SE, Hsieh K, Donahue P, et al. Could complementary health approaches improve the symptom experience and outcomes of critically ill adults? A systematic review of randomized controlled trials.[J]. Complement Ther Med, 2019, 47:102166.
34. [Pandharipande](https://pubmed.ncbi.nlm.nih.gov/?term=Pandharipande+PP&cauthor_id=24401069) PP, [Girard](https://pubmed.ncbi.nlm.nih.gov/?term=Girard+TD&cauthor_id=24401069) TD, [Ely](https://pubmed.ncbi.nlm.nih.gov/?term=Ely+EW&cauthor_id=24401069) EW. Long-term Cognitive Impairment After Critical Illness.[J]. N Engl J Med, 2014, 370(2):185-186.
35. Neto AS, Nassar AP, Cardoso SO, et al. Delirium screening in critically ill patients: a systematic review and meta-analysis[J]. Critical Care, 2012, 40(6):1946-1951.
36. Smith J, Gearhart E, Djurkovic S. [Ketamine continuous infusion for analgosedation in critically ill mechanically ventilated adults](https://www-embase-com.ezproxy.lb.polyu.edu.hk/a/#/search/results?subaction=viewrecord&rid=46&page=1&id=L613520415).[J]. Crit Care Med, 2016, 44(Suppl 1):289.
37. Scruth E, Marelich G, Cheng E, et al. [Large enterprise multidisciplinary solution to reducing variability in care in the ICU](https://www-embase-com.ezproxy.lb.polyu.edu.hk/a/#/search/results?subaction=viewrecord&rid=47&page=1&id=L613520588).[J]. Crit Care Med, 2016, 44(Suppl 1):366.
38. Morandi A, Brummel NE, Ely EW. [Sedation, delirium and mechanical ventilation: The 'ABCDE' approach](https://www-embase-com.ezproxy.lb.polyu.edu.hk/a/#/search/results?subaction=viewrecord&rid=100&page=1&id=L51197083).[J]. Curr Opin Crit Care, 2011, 17(1):43-49.
39. Riker RR, Fraser GL, Cox PM. [Continuous infusion of haloperidol controls agitation in critically ill patients](https://www-embase-com.ezproxy.lb.polyu.edu.hk/a/#/search/results?subaction=viewrecord&rid=112&page=1&id=L24085295).[J]. Crit Care Med, 1994, 22(3):433-440.
40. Pochard F, Lanore JJ, Bellivier F, et al. Subjective psychological status of severely ill patients discharged from mechanical ventilation.[J]. Clin Intensive Care, 1995, 6(2):57-61.
41. Zaal I J, Devlin JW, Peelen LM, et al. A Systematic Review of Risk Factors for Delirium in the ICU*[J]. Critical Care Medicine, 2015, 43(1):40-47.
42. Burry LD, Williamson DR, Perreault MM, et al. Analgesic, sedative, antipsychotic, and neuromuscular blocker use in Canadian intensive care units: a prospective, multicentre, observational study.[J]. Can J Anaesth, 2014, 61(7):619-630.
43. Fraser D, Spiva L, Forman W, et al. Original Research: Implementation of an Early Mobility Program in an ICU.[J]. Am J Nurs, 2015, 115(12):49-58.
44. McAndrew NS, Leske J, Guttormson J, et al. Quiet time for mechanically ventilated patients in the medical intensive care unit.[J]. Intensive Crit Care Nurs, 2016, 35:22-27.
45. Almeida TM, Azevedo LC, [Nosé](https://pubmed.ncbi.nlm.nih.gov/?term=Nos%C3%A9+PM&cauthor_id=28099638) PM, et al. Risk factors for agitation in critically ill patients.[J]. Rev Bras Ter Intensiva, 2016, 28(4):413-419.
46. Liu V, Herbert D, Foss-Durant A, et al. Evaluation Following Staggered Implementation of the "Rethinking Critical Care" ICU Care Bundle in a Multicenter Community Setting.[J]. Crit Care Med, 2016, 44(3):460-467.
47. Castillo MI, Cooke M, Macfarlane B, et al. Factors associated with anxiety in critically ill patients: A prospective observational cohort study.[J]. Int J Nurs Stud, 2016, 60:225-233.
48. Fuller BM, Roberts BW, Mohr NM, et al. The ED-SED Study: A Multicenter, Prospective Cohort Study of Practice Patterns and Clinical Outcomes Associated With Emergency Department SEDation for Mechanically Ventilated Patients.[J]. Crit Care Med, 2019, 47(11):1539-1548.
49. Wilson JE, Carlson R, Duggan MC, et al. Delirium and Catatonia in Critically Ill Patients: The Delirium and Catatonia Prospective Cohort Investigation.[J]. Crit Care Med, 2017, 45(11):1837-1844.
50. Soja SL, Pandharipande PP, Fleming SB, et al. Implementation, reliability testing, and compliance monitoring of the Confusion Assessment Method for the Intensive Care Unit in trauma patients.[J]. Intensive Care Med, 2008,34(7):1263-1268.

Patients failed to meet the inclusion criteria

1. Huber FT, Bartels H, Siewert JR.Treatment of postoperative alcohol withdrawal syndrome after esophageal resection.[J]. Langenbecks Arch Chir Suppl II Verh Dtsch Ges Chir. 1990:1141-1143.
2. Gold JA, Rimal B, Nolan A, et al. A strategy of escalating doses of benzodiazepines and phenobarbital administration reduces the need for mechanical ventilation in delirium tremens.[J]. Crit Care Med, 2007, 35(3):724-730.
3. Lucidarme O, Seguin A, Daubin C, et al. Nicotine withdrawal and agitation in ventilated critically ill patients.[J]. Crit Care, 2010, 14(2):R58.
4. Isik B, Arslan M, Tunga AD, et al. Dexmedetomidine decreases emergence agitation in pediatric patients after sevoflurane anesthesia without surgery[J]. Pediatric Anesthesia, 2006, 16(7):748-753.
5. Viitanen H, Annila P, Rorarius M, et al. Recovery after halothane anaesthesia induced with thiopental, propofol-alfentanil or halothane for day-case adenoidectomy in small children.[J]. Br J Anaesth, 1998, 81(6):960-962.
6. DeMuro JP, Botros DG, Wirkowski E, et al. Use of dexmedetomidine for the treatment of alcohol withdrawal syndrome in critically ill patients: a retrospective case series.[J]. J Anesth, 2012, 26(4):601-605.
7. Duby JJ, Berry AJ, Ghayyem P, et al. Alcohol withdrawal syndrome in critically ill patients: protocolized versus nonprotocolized management.[J]. J Trauma Acute Care Surg, 2014, 77(6):938-943.
8. Sohraby R, Attridge RL, Hughes DW. Use of propofol-containing versus benzodiazepine regimens for alcohol withdrawal requiring mechanical ventilation.[J]. Ann Pharmacother, 2014, 48(4):456-461.
9. Luetz A, Gensel D, [Müller](https://pubmed.ncbi.nlm.nih.gov/?term=M%C3%BCller+J&cauthor_id=27276343) J, et al. Validity of Different Delirium Assessment Tools for Critically Ill Children: Covariates Matter.[J]. Crit Care Med, 2016, 44(11):2060-2069.
10. Stewart R, Perez R, Musial B, et al. Outcomes of Patients with Alcohol Withdrawal Syndrome Treated with High-Dose Sedatives and Deferred Intubation.[J]. Ann Am Thorac Soc, 2016, 13(2):248-252.
11. Neubert A, Baarslag MA, Dijk MV, et al. The CLOSED trial; CLOnidine compared with midazolam for SEDation of paediatric patients in the intensive care unit: study protocol for a multicentre randomised controlled trial.[J]. BMJ Open, 2017, 7(6):e016031.
12. Kiski D, Malec E, Schmidt C. Use of dexmedetomidine in pediatric cardiac anesthesia.[J]. Curr Opin Anaesthesiol, 2019, 32(3):334-342.
13. He S, Wang YL, Zuo ZL. Clinical application of the Chinese version of Cornell assessment of pediatric delirium: a pilot study.[J]. Zhonghua Er Ke Za Zhi, 2019, 57(5):344-349.
14. Kim MS, Moon BE, Kim H, et al. Comparison of propofol and fentanyl administered at the end of anesthesia for prevention of emergence agitation after sevoflurane anaesthesia in children.[J]. Br J Anaesth, 2013,110(2):274-280.
15. Wolfe KS, Patel BK, Pohlman AS, et al. [Delirium and sedative requirements in a trial of helmet versus facemask noninvasive ventilation in acute hypoxic respiratory failure](https://www-embase-com.ezproxy.lb.polyu.edu.hk/a/#/search/results?subaction=viewrecord&rid=45&page=1&id=L617706432).[J]. Am J Respir Crit Care Med, 2017, 195.
16. Barnes-Daly MA, Balas M, Phillips G, [The effect of sedation scores on compliance with the ABCDEF bundle in seven community hospital ICUs](https://www-embase-com.ezproxy.lb.polyu.edu.hk/a/#/search/results?subaction=viewrecord&rid=50&page=1&id=L613522573).[J]. Crit Care Med, 2016, 44(Suppl 1):397.
17. El Shora H, Amr Y, El Sherief S.[Prospective study on the effect of inhalational versus intravenous sedation on delirium and agitation in patients admitted to ICU postcardiac surgery](https://www-embase-com.ezproxy.lb.polyu.edu.hk/a/#/search/results?subaction=viewrecord&rid=66&page=1&id=L72329063).[J]. J Cardiothorac Vasc Anesth, 2016, 30(Suppl 1):S34.
18. Abdelgalel EF.[Dexmedetomidine versus haloperidol for prevention of delirium during non-invasive mechanical ventilation](https://www-embase-com.ezproxy.lb.polyu.edu.hk/a/#/search/results?subaction=viewrecord&rid=56&page=1&id=L613480560).[J]. Egypt J Anaesth, 2016, 32(4):473-481.
19. Yapici N, Coruh T, Kehlibar T, et al. [Dexmedetomidine in cardiac surgery patients who fail extubation and present with a delirium state](https://www-embase-com.ezproxy.lb.polyu.edu.hk/a/#/search/results?subaction=viewrecord&rid=98&page=1&id=L361696760).[J]. Heart Surg Forum, 2011, 14(2):E93-E98.
20. Shukry M, Clyde MC, Kalarickal PL, et al. Does dexmedetomidine prevent emergence delirium in children after sevoflurane-based general anesthesia?[J]. Pediatric Anesthesia, 2005, 15(12):1098-1104.
21. Thomason JW, Shintani A, Peterson JF, et al. Intensive care unit delirium is an independent predictor of longer hospital stay: a prospective analysis of 261 non-ventilated patients.[J]. Crit Care, 2005, 9(4):R375-381.
22. Seymour CW, Pandharipande PP, Koestner T, et al. Diurnal sedative changes during intensive care: impact on liberation from mechanical ventilation and delirium.[J]. Crit Care Med, 2012, 40(10):2788-2796.
23. Awissi DK, [Bégin](https://pubmed.ncbi.nlm.nih.gov/?term=B%C3%A9gin+C&cauthor_id=22202496) C, Moisan J, et al. I-SAVE study: impact of sedation, analgesia, and delirium protocols evaluated in the intensive care unit: an economic evaluation.[J]. Ann Pharmacother, 2012, 46(1):21-28.
24. Aydogan MS, Korkmaz MF, [Ozgül](https://pubmed.ncbi.nlm.nih.gov/?term=Ozg%C3%BCl+U&cauthor_id=23448434) U, et al. Pain, fentanyl consumption, and delirium in adolescents after scoliosis surgery: dexmedetomidine vs midazolam.[J]. Paediatr Anaesth, 2013, 23(5):446-452.
25. Michaud CJ, Bullard HM, Harris SA, et al. Impact of Quetiapine Treatment on Duration of Hypoactive Delirium in Critically Ill Adults: A Retrospective Analysis.[J]. Pharmacotherapy, 2015, 35(8):731-739.
26. Al-Qadheeb NS, Skrobik Y, Schumaker G, et al. Preventing ICU Subsyndromal Delirium Conversion to Delirium With Low-Dose IV Haloperidol: A Double-Blind, Placebo-Controlled Pilot Study.[J]. Crit Care Med, 2016, 44(3):583-591.
27. Peterson JF, Pun BT, Dittus RS, et al. Delirium and Its Motoric Subtypes: A Study of 614 Critically Ill Patients.[J]. J Am Geriatr Soc, 2006, 54(3):479-484.
28. Curley MAQ, Watson RS, Cassidy AM, et al. Design and rationale of the "Sedation strategy and cognitive outcome after critical illness in early childhood" study.[J]. Contemp Clin Trials, 2018, 72:8-15.
29. Louis C, Godet T, Chanques G, et al. Effects of dexmedetomidine on delirium duration of non-intubated ICU patients (4D trial): study protocol for a randomized trial.[J]. Trials, 2018, 19(1):307.
30. Singh TD, O'Horo JC, Gajic O, et al. Risk factors and outcomes of critically ill patients with acute brain failure: A novel end point.[J]. J Crit Care. 2018, 43:42-47.
31. Akin A, Esmaoglu A, Guler G, et al. Propofol and Propofol–Ketamine in Pediatric Patients Undergoing Cardiac Catheterization[J]. pediatric cardiology, 2005, 26(5):553-557.
32. La MK, Thompson Bastin ML, Gisewhite JT, et al. Impact of restarting home neuropsychiatric medications on sedation outcomes in medical intensive care unit patients.[J].J Crit Care,2018, 43:102-107.
33. Vourc’h M, Feuillet F, Mahe PJ, et al. Baclofen to prevent agitation in alcohol-addicted patients in the ICU: study protocol for a randomised controlled trial.[J]. Trials, 2016, 17(1):415.

Intervention failed to meet the inclusion criteria

1. Hendrie K, Khan S, Perkins A, et al.[Incidence and prevalence of delirium in the intensive care unit over time: A longitudinal study](https://www-embase-com.ezproxy.lb.polyu.edu.hk/a/#/search/results?subaction=viewrecord&rid=10&page=1&id=L629628732).[J]. Crit Care Med, 2019, 47(Suppl 1).
2. Soliman M, Radwan T, Osman S, et al. [The effect of melatonin administration on sedation level as adjuvant to propofol in traumatic brain injury patients](https://www-embase-com.ezproxy.lb.polyu.edu.hk/a/#/search/results?subaction=viewrecord&rid=16&page=1&id=L624863930).[J]. Intensive Care Med Exp, 2018, 6(Suppl 2).
3. Lavrentieva A, Papaioannou M, Tsloulis I, er al. [Efficacy of dexmedetomidine in reducing duration of mechanical ventilation in patients with severe burn injury](https://www-embase-com.ezproxy.lb.polyu.edu.hk/a/#/search/results?subaction=viewrecord&rid=17&page=1&id=L624864129).[J]. Intensive Care Med Exp, 2018, 6(Suppl 2).
4. Sato K, Taniguchi T, Okajima M. [Dexmedetomidine for delirium prevention in nonsurgical cardiac patients on mechanical life support](https://www-embase-com.ezproxy.lb.polyu.edu.hk/a/#/search/results?subaction=viewrecord&rid=30&page=1&id=L620080704).[J]. Crit Care Med, 2018, 46(Suppl 1):348.
5. Fuchita M, Perkins A, Khan S, et al. [Perioperative risk factors for postoperative delirium in patients undergoing esophagectomy](https://www-embase-com.ezproxy.lb.polyu.edu.hk/a/#/search/results?subaction=viewrecord&rid=32&page=1&id=L620080862).[J]. Crit Care Med, 2018, 46(Suppl 1):369.
6. Thille AW, Reynaud F, Barrau S, et al. [Impact of sleep quality on duration of weaning from mechanical ventilation](https://www-embase-com.ezproxy.lb.polyu.edu.hk/a/#/search/results?subaction=viewrecord&rid=40&page=1&id=L619043303).[J]. Intensive Care Med Exp, 2017, 5(Suppl 1).
7. Benzodiazepine-associated delirium in critically ill adults[J]. Intensive Care Medicine, 2015, 41(12):2130-2137.
8. Mcpherson J A , Wagner C E , Boehm L M , et al. Delirium in the Cardiovascular ICU: Exploring Modifiable Risk Factors[J]. Critical care medicine, 2012, 41(2):405-413.
9. Fossat G, Baudin F, Coulanges C, et al. [Electrical muscle stimulation and bicycling combined to early standard rehabilitation versus early standard rehabilitation alone: Impact on global muscle strength at ICU discharge-an open-label, single-centre, assessor-blinded randomised trial](https://www-embase-com.ezproxy.lb.polyu.edu.hk/a/#/search/results?subaction=viewrecord&rid=42&page=1&id=L614625743).[J]. Ann Intensive Care, 2017, 7(Suppl 1):9.
10. Mackenzie E, Wolfe KS, Patel S, et al. [Relationship between mean arterial pressure and delirium in mechanically ventilated patients](https://www-embase-com.ezproxy.lb.polyu.edu.hk/a/#/search/results?subaction=viewrecord&rid=44&page=1&id=L617704076).[J]. Am J Respir Crit Care Med, 2017, 195.
11. Ely EW, Inouye SK, Bernard GR, et al.Delirium in Mechanically Ventilated Patients: Validity and Reliability of the Confusion Assessment Method for the Intensive Care Unit (CAM-ICU).[J]. JAMA, 286(21):2703-2710.
12. Nunes Sl, Forsberg S, Blomqvist H, et al. [Effect of dexmedetomidine on weaning from mechanical ventilation in intensive care patients](https://www-embase-com.ezproxy.lb.polyu.edu.hk/a/#/search/results?subaction=viewrecord&rid=58&page=1&id=L617955203).[J]. Intensive Care Med Exp, 2016, 4(Suppl 1).
13. Louro J, Ray JJ, Ruiz XD, et al. [A prospective observational pilot study of ICU sedation variation using bispectral index to identify diurnal patterns related to change of nursing shifts](https://www-embase-com.ezproxy.lb.polyu.edu.hk/a/#/search/results?subaction=viewrecord&rid=65&page=1&id=L613553315).[J]. Anesth Analg, 2016, 122(Suppl 3):S91.
14. Maldonado JR, Wysong A, Starre PJA, et al. Dexmedetomidine and the Reduction of Postoperative Delirium After Cardiac Surgery.[J]. Psychosomatics, 2009, 50(3):206-217.
15. Hsieh SJ, Fuster D, D'Alessandro DA, [Feasibility and efficacy of intranasal insulin for post-operative delirium: The CNS-elders randomized controlled trial](https://www-embase-com.ezproxy.lb.polyu.edu.hk/a/#/search/results?subaction=viewrecord&rid=77&page=1&id=L72051893).[J]. Am J Respir Crit Care Med, 2015, 191.
16. Milbrandt EB, Kersten A, Kong L, et al. Haloperidol use is associated with lower hospital mortality in mechanically ventilated patients.[J]. Crit Care Med, 2005, 33(1):226-229.
17. Masica AL, Girard TD, Wilkinson GR, et al. Clinical sedation scores as indicators of sedative and analgesic drug exposure in intensive care unit patients.[J]. Am J Geriatr Pharmacother, 20075(3):218-231.
18. Pandharipande P, Cotton BA, Shintani A, et al. Prevalence and risk factors for development of delirium in surgical and trauma intensive care unit patients.[J]. J Trauma, 2008, 65(1):34-41.
19. Pohlman MC, Schweickert WD, Pohlman AS, et al. Feasibility of physical and occupational therapy beginning from initiation of mechanical ventilation.[J]. Crit Care Med, 2010, 38(11):2089-2094.
20. Zhao D, Xu Y, He W, et al. A comparison of bispectral index and sedation agitation scale in guiding sedation therapy: a randomized controlled study in patients undergoing short term mechanical ventilation.[J]. Zhongguo Wei Zhong Bing Ji Jiu Yi Xue. 2011, 23(4):220-223.
21. McGrane S, Girard TD, Thompson JL, et al. Procalcitonin and C-reactive protein levels at admission as predictors of duration of acute brain dysfunction in critically ill patients.[J]. Crit Care, 2011, 15(2):R78.
22. Robinson BR, Blakeman TC, Toth P, et al. Patient-ventilator asynchrony in a traumatically injured population.[J]. Respir Care, 2013, 58(11):1847-1855.
23. Wan LJ, Huang QQ, Yue JX, et al. Comparison of sedative effect of dexmedetomidine and midazolam for post-operative patients undergoing mechanical ventilation in surgical intensive care unit.[J]. Zhongguo Wei Zhong Bing Ji Jiu Yi Xue, 2011, 23(9):543-546.
24. Mendez-Tellez PA, Dinglas VD, Colantuoni E, et al. Factors associated with timing of initiation of physical therapy in patients with acute lung injury.[J]. J Crit Care, 2013, 28(6):980-984.
25. Nilsen ML, Sereika S, Happ MB. Nurse and patient characteristics associated with duration of nurse talk during patient encounters in ICU.[J]. Heart Lung, 2013, 42(1):5-12.
26. Huang F, Wang J, Yang X, et al. Sedative effects of dexmedetomidine in post-operative elder patients on mechanical ventilation.[J]. Zhonghua Yi Xue Za Zhi, 2014, 94(41):3211-3215.
27. Simonis FD, Binnekade JM, Braber A, et al. PReVENT--protective ventilation in patients without ARDS at start of ventilation: study protocol for a randomized controlled trial.[J]. Trials, 2015, 16:226.
28. Song R, Li J, Dong C, et al. A study of using dexmedetomidine in ventilator bundle treatment in an ICU.[J]. Zhonghua Wei Zhong Bing Ji Jiu Yi Xue, 2015, 27(10):836-840.
29. Lyu J, Liu D, An Y, et al. The influence of the sedation based on remifentanil analgesia on the occurrence of delirium in critically ill patients.[J]. Zhonghua Wei Zhong Bing Ji Jiu Yi Xue, 2015, 27(10):845-849.
30. Eremenko AA, Chemova EV. Comparison of dexmedetomidine and propofol for short-term sedation in early postoperative period after cardiac surgery.[J]. Anesteziol Reanimatol, 2014, (2):37-41.
31. Kamdar BB, Combs MP, Colantuoni E, et al. The association of sleep quality, delirium, and sedation status with daily participation in physical therapy in the ICU.[J]. Crit Care, 2016, 19:261.
32. Bergeron N, Dubois MJ, Dumont M, et al. Intensive Care Delirium Screening Checklist: Evaluation of a New Screening Tool.[J]. ensive Care Med, 2001, 27(5):859-864.
33. Guenther U, Koegl F, Theuerkauf N, et al. Nursing workload indices TISS-10, TISS-28, and NEMS : Higher workload with agitation and delirium is not reflected.[J]. Med Klin Intensivmed Notfmed, 2016, 111(1):57-64.
34. Lu X, Li J, Li T, et al. Clinical study of midazolam sequential with dexmedetomidine for agitated patients undergoing weaning to implement light sedation in intensive care unit.[J]. Chin J Traumatol, 2016, 19(2):94-96.
35. Li J, Dong C, Zhang H, et al. Study of prevention and control of delirium in ventilated patients by simulating blockage of circadian rhythm with sedative in intensive care unit.[J]. Zhonghua Wei Zhong Bing Ji Jiu Yi Xue, 2016, 28(1):50-56.
36. Boesen HC, Andersen JH, Bendtsen AO, et al. Sleep and delirium in unsedated patients in the intensive care unit.[J]. Acta Anaesthesiol Scand, 2016, 60(1):59-68.
37. Yu HJ, Xu XQ, Xu SA, et al. Analgesic and Sedative Effect of Acupuncture Combined with Medicine on Patients Undergiong Cardiac Surgery.[J]. Zhongguo Zhong Xi Yi Jie He Za Zhi, 2016, 36(3):289-293.
38. Piriyapatsom A, Chittawatanarat K, Kongsayreepong S, et al. Incidence and Risk Factors of Unplanned Extubation in Critically Ill Surgical Patients: The Multi-center Thai University-based Surgical Intensive Care Units Study (THAI-SICU Study).[J]. J Med Assoc Thai, 2016, 99(Suppl 6):S153-S162.
39. Levanen J, Makela ML, Scheinin H. Dexmedetomidine Premedication Attenuates Ketamine-induced Cardiostimulatory Effects and Postanesthetic Delirium[J]. Anesthesiology, 1995, 82(5):1117-1125.
40. Nadler JW, Evans JL, Fang E, et al. A randomised trial of peri-operative positive airway pressure for postoperative delirium in patients at risk for obstructive sleep apnoea after regional anaesthesia with sedation or general anaesthesia for joint arthroplasty.[J]. Anaesthesia, 2017, 72(6):729-736.
41. Shao C, Gu L, Mei Y, et al. Analysis of the risk factors of cognitive impairment in post-intensive care syndrome patient.[J]. Zhonghua Wei Zhong Bing Ji Jiu Yi Xue, 2017, 29(8):716-720.
42. Liu X, Lyu J, An Y. Explore objective clinical variables for detecting delirium in ICU patients: a prospective case-control study.[J]. Zhonghua Wei Zhong Bing Ji Jiu Yi Xue, 2017, 29(4):347-352.
43. Girard TD, Exline MC, Carson SS, et al. Haloperidol and Ziprasidone for Treatment of Delirium in Critical Illness.[J]. N Engl J Med, 2018, 379(26):2506-2516.
44. Avrami S, Argyriou G, Kadda O, et al. Delirium in Intensive Care Unit. Factors that affect the appearance of delirium and its importance to the patients' final outcome.[J]. To VimaI Tou Asklipiou, 2012, 11(4):549-562.
45. Pun BT, Balas MC, Barnes-Daly MA, et al. Caring for Critically Ill Patients with the ABCDEF Bundle: Results of the ICU Liberation Collaborative in Over 15,000 Adults.[J]. Crit Care Med, 2019, 47(1):3-14.
46. Su L, Yan Y, Huang W, et al. Risk factors for intensive care unit delirium after cardiac operation.[J]. Zhonghua Wei Zhong Bing Ji Jiu Yi Xue, 2019, 31(2):165-171.
47. Eremenko AA, Chernova EV. Dexmedetomidine use for intravenous sedation and delirium treatment during early postoperative period in cardio-surgical patients.[J]. Anesteziol Reanimatol, 2013, (5):4-8.
48. Torres-Contreras CC, [Páez-Esteban](https://pubmed.ncbi.nlm.nih.gov/?term=P%C3%A1ez-Esteban+AN&cauthor_id=29909126) AN, [Hinestrosa-Díaz Del Castillo](https://pubmed.ncbi.nlm.nih.gov/?term=Hinestrosa-D%C3%ADaz+Del+Castillo+A&cauthor_id=29909126), et al. Factors associated with delirium in critical patients in a health institution in Bucaramanga, Colombia.[J]. Enferm Intensiva, 2019, 30(1):13-20.
49. Ritchie BM, Torbic H, DeGrado JR, et al. Sedation Variability Increases Incidence of Delirium in Adult Medical Intensive Care Unit Patients at a Tertiary Academic Medical Center.[J]. Am J Ther, 2019, 26(1):e92-e95.
50. Corbett SM, Rebuck JA, Greene CM, et al. Dexmedetomidine does not improve patient satisfaction when compared with propofol during mechanical ventilation.[J]. Crit Care Med, 2005, 33(5):940-945.
51. Muellejans B, Matthey T, Scholpp J, et al. Sedation in the intensive care unit with remifentanil/propofol versus midazolam/fentanyl: a randomised, open-label, pharmacoeconomic trial.[J]. Crit Care, 2006, 10(3):R91.
52. Dessap AM, Roche-Campo F, Launay JM, et al. Delirium and Circadian Rhythm of Melatonin During Weaning From Mechanical Ventilation: an Ancillary Study of a Weaning Trial.[J]. Chest, 2015,148(5):1231-1241.
53. Stollings JL, Thompson JL, Ferrell BA, et al. Sedative Plasma Concentrations and Delirium Risk in Critical Illness.[J]. Ann Pharmacother, 2018, 52(6):513-521.
54. Meagher DJ, Leonard M, Donnelly S, et al. A longitudinal study of motor subtypes in delirium: Relationship with other phenomenology, etiology, medication exposure and prognosis[J]. journal of psychosomatic research, 2011, 71(6):395-403.
55. Chabanne R, Perbet S, Futier E, et al. Impact of the Anesthetic Conserving Device on Respiratory Parameters and Work of Breathing in Critically Ill Patients Under Light Sedation With Sevoflurane.[J]. Anesthesiology, 2014, 121(4):808-816.
56. Castano Gamboa N, Reyes J, Ruiz F. [PDG13 cost effectiveness analysis of dexmetomidine compared with propofol and midazolam for mechanically ventilated adult patients in colombia.[](https://www-embase-com.ezproxy.lb.polyu.edu.hk/a/#/search/results?subaction=viewrecord&rid=5&page=1&id=L2002156653)J]. Value Health, 2019, 22(Suppl 2):S165.
57. Ben-Rehouma M, Moulin C, Montravers P. [The usefulness of dexmedetomidine after lung transplantation in intensive care unit](https://www-embase-com.ezproxy.lb.polyu.edu.hk/a/#/search/results?subaction=viewrecord&rid=34&page=1&id=L621461679).[J]. Crit Care, 2018, 22(Suppl 1).
58. Kaila M, Everingham K, Lapinlampi P, et al. A randomized controlled proof-of-concept trial of early sedation management using responsiveness Index monitoring in mechanically ventilated critically ill patients.[J]. Crit Care, 2015, 19(1):333.

Outcomes failed to meet the inclusion criteria

1. Sich N, Schulingkamp D, Bertozzi D, et al. [Sedation administration timing: Intermittent dosing reduces time to extubation (satire trial)](https://www-embase-com.ezproxy.lb.polyu.edu.hk/a/#/search/results?subaction=viewrecord&rid=29&page=1&id=L620080550).[J]. Crit Care Med, 2018, 46(Suppl 1):9.
2. Lazim R, Knauth M, Allmond K. [Improving utilization of fentanyl boluses for ventilated patients using smart pump technology](https://www-embase-com.ezproxy.lb.polyu.edu.hk/a/#/search/results?subaction=viewrecord&rid=33&page=1&id=L620081026).[J]. Crit Care Med, 2018, 46(Suppl 1):581.
3. Yang S, Liu J, Yang W, et al. Effect of early mobilization on diaphragmatic function in patients with mechanical ventilation: a prospective randomized controlled study.[J]. Zhonghua Wei Zhong Bing Ji Jiu Yi Xue, 2018, 30(2):112-116.
4. Liu X, Zhang K, Wang W, et al. Dexmedetomidine Versus Propofol Sedation Improves Sublingual Microcirculation After Cardiac Surgery: a Randomized Controlled Trial.[J]. J Cardiothorac Vasc Anesth, 2016, 30(6):1509-1515.
5. Shurtleff V, Radosevich J, Patanwala A. [Delirium and coma-free days in patients receiving continuous-infusion ketamine versus propofol](https://www-embase-com.ezproxy.lb.polyu.edu.hk/a/#/search/results?subaction=viewrecord&rid=27&page=1&id=L620080375).[J]. Crit Care Med, 2018, 46(Suppl 1):462.

No intervention

1. Pollack L, Caldwell E, Ungar A, et al. [Feasibility and reliability of a simplified bedside strength assessment in critically ill patients](https://www-embase-com.ezproxy.lb.polyu.edu.hk/a/#/search/results?subaction=viewrecord&rid=8&page=1&id=L630355680).[J]. Am J Respir Crit Care Med, 2019, 199(9).
2. Wilson J, Ely E, Dittus R, et al. [Delirium and catatonia in critically ill patients: The decat prospective cohort investigation](https://www-embase-com.ezproxy.lb.polyu.edu.hk/a/#/search/results?subaction=viewrecord&rid=26&page=1&id=L620080201).[J]. Crit Care Med, 2018, 46(Suppl 1):212.
3. Brophy A, Cardinale M, Andrews LB, et al. [Prospective evaluation of pain and agitation practices across New Jersey intensive care units](https://www-embase-com.ezproxy.lb.polyu.edu.hk/a/#/search/results?subaction=viewrecord&rid=49&page=1&id=L613520775).[J]. Crit Care Med, 2016, 44(Suppl 1):302.
4. Vyveganathan L, Izaham A, Wan R,  et al. Delirium in critically ill patients: incidence, risk factors and outcomes.[J]. Crit Care Shock, 2019, 22(1):25-40.
5. Jayaswal A, Sampath H, Soohinda G, et al. Delirium in medical intensive care units: Incidence, subtypes, risk factors, and outcome.[J]. Indian J Psychiatry, 2019, 61(4):352-358.
6. Von Rueden KT, Wallizer B, Thurman P, et al. Delirium in Trauma Patients: Prevalence and Predictors.[J]. Crit Care Nurse, 2017, 37(1):40-48.
7. Tilouche N, Hassen MF, Ali HBS, et al. Delirium in the Intensive Care Unit: Incidence, Risk Factors, and Impact on Outcome.[J]. Indian J Crit Care Med, 2018, 22(3):144-149.
8. Shehabi Y, Nakae H, Hammond N, et al. The effect of dexmedetomidine on agitation during weaning of mechanical ventilation in critically ill patients.[J]. Anaesth Intensive Care, 2010, 38(1):82-90.
9. Puntillo KA, Arai S, Cohen NH, et al. Symptoms experienced by intensive care unit patients at high risk of dying.[J]. Crit Care Med, 2010, 38(11):2155-2160.
10. Tsuruta R, Oda Y, Shintani A, et al. Delirium and coma evaluated in mechanically ventilated patients in the intensive care unit in Japan: a multi-institutional prospective observational study.[J]. J Crit Care, 2014, 29(3):472.e1-5.
11. Andresen JM, Girard TD, Pandharipande PP, et al. Burst suppression on processed electroencephalography as a predictor of postcoma delirium in mechanically ventilated ICU patients.[J]. Crit Care Med, 2014, 42(10):2244-2251.
12. Cole JB, Klein LR, Nystrom PC, et al. A prospective study of ketamine as primary therapy for prehospital profound agitation.[J]. Am J Emerg Med, 2018, 36(5):789-796.

**STEP 3: 105 full text articles excluded**

Intervention not sedation depth

1. Azeem TMA, Yosif NE, Alansary AM, et al. [Dexmedetomidine vs morphine and midazolam in the prevention and treatment of delirium after adult cardiac surgery; A randomized, double-blinded clinical trial](https://www-embase-com.ezproxy.lb.polyu.edu.hk/a/#/search/results?subaction=viewrecord&rid=22&page=1&id=L622935321).[J]. Saudi J Anaesth, 2018,12(2):190-197.
2. Vijayakumar HN, Ramya K, Duggappa DR, et al. [Effect of melatonin on duration of delirium in organophosphorus compound poisoning patients: A double-blind randomised placebo controlled trial](https://www-embase-com.ezproxy.lb.polyu.edu.hk/a/#/search/results?subaction=viewrecord&rid=1&page=1&id=L613239014).[J].Indian J Anaesth, 2016 60(11):814-820.
3. Luo J, Wang MY, Liang BM, et al. I[nitial synchronized intermittent mandatory ventilation versus assist/control ventilation in treatment of moderate acute respiratory distress syndrome: A prospective randomized controlled trial](https://www-embase-com.ezproxy.lb.polyu.edu.hk/a/#/search/results?subaction=viewrecord&rid=83&page=1&id=L607740838).[J]. J Thorac Dis, 2015,7(12):2262-2273.
4. Ingalls NK, Armstrong B, Hester M, et al. The Fog of War: Delirium Prevalence in a Combat Intensive Care Unitil Med.[J]. Milit Med, 2016, 181(3):209-212.
5. Lachaine J, Beauchemin C. Economic evaluation of dexmedetomidine relative to midazolam for sedation in the intensive care unit.[J]. Can J Hosp Pharm, 2012, 65(2):103-110.
6. Granberg Axell AI, Malmros CW, Bergbom IL, et al. Intensive care unit syndrome/delirium is associated with anemia, drug therapy and duration of ventilation treatment.[J]. Acta Anaesthesiol Scand, 2002, 46(6):726-731.
7. Ely EW, Shintani A, Truman B, et al. Delirium as a predictor of mortality in mechanically ventilated patients in the intensive care unit.[J]. JAMA, 2004, 291(14):1753-1762.
8. Ely EW, Truman B, Manzi DJ, et al. Consciousness monitoring in ventilated patients: bispectral EEG monitors arousal not delirium.[J]. Intensive Care Med, 2004, 30(8):1537-1543.
9. Pun BT, Gordon SM, Peterson JF, et al. Large-scale implementation of sedation and delirium monitoring in the intensive care unit: a report from two medical centers.[J]. Crit Care Med, 2005, 33(6):1199-1205.
10. Pandharipande P, Cotton BA, Shintani A, et al. Motoric subtypes of delirium in mechanically ventilated surgical and trauma intensive care unit patients.[J]. Intensive Care Med, 2007, 33(10):1726-1731.
11. Watson PL, Shintani AK, Tyson R, et al. Presence of electroencephalogram burst suppression in sedated, critically ill patients is associated with increased mortality.[J]. Crit Care Med, 2008, 36(12):3171-3177.
12. Riker RR, Shehabi Y, Bokesch PM, et al. Dexmedetomidine vs midazolam for sedation of critically ill patients: a randomized trial.[J]. JAMA, 2009, 301(5):489-499.
13. Balas MC, Happ MB, Yang W, et al. Outcomes Associated With Delirium in Older Patients in Surgical ICUs.[J]. Chest, 2009, 135(1):18-25.
14. Reade MC, O'Sullivan K, Bates S, et al. Dexmedetomidine vs. haloperidol in delirious, agitated, intubated patients: a randomised open-label trial.[J]. Crit Care. 2009, 13(3):R75.
15. Pandharipande PP, Sanders RD, Girard TD, et al. Effect of dexmedetomidine versus lorazepam on outcome in patients with sepsis: an a priori-designed analysis of the MENDS randomized controlled trial.[J]. Crit Care, 2010, 14(2):R38.
16. Shehabi Y, Riker RR, Bokesch PM, et al. Delirium duration and mortality in lightly sedated, mechanically ventilated intensive care patients.[J]. Crit Care Med, 2010, 38(12):2311-2318.
17. Girard TD, Pandharipande PP, Carson SS, et al. Feasibility, efficacy, and safety of antipsychotics for intensive care unit delirium: the MIND randomized, placebo-controlled trial.[J]. Crit Care Med, 2010, 38(2):428-437.
18. Skrobik Y, Ahern S, Leblanc M, et al. Protocolized intensive care unit management of analgesia, sedation, and delirium improves analgesia and subsyndromal delirium rates.[J]. Anesth Analg, 2010, 111(2):451-463.
19. Stransky M, Schmidt C, Ganslmeier P, et al. Hypoactive delirium after cardiac surgery as an independent risk factor for prolonged mechanical ventilation.[J]. J Cardiothorac Vasc Anesth, 2011, 25(6):968-974.
20. Mehta S, Burry L, Cook D, et al. Daily sedation interruption in mechanically ventilated critically ill patients cared for with a sedation protocol: a randomized controlled trial.[J]. JAMA, 2012, 308(19):1985-1992.
21. Takeuchi M, Takeuchi H, Fujisawa D, et al. Incidence and risk factors of postoperative delirium in patients with esophageal cancer.[J]. Ann Surg Oncol, 2012, 19(12):3963-3970.
22. Tomasi CD, Grandi C, Salluh J, et al. Comparison of CAM-ICU and ICDSC for the detection of delirium in critically ill patients focusing on relevant clinical outcomes.[J]. J Crit Care, 2012, 27(2):212-217.
23. Dale CR, Bryson CL, Fan VS, et al. A greater analgesia, sedation, delirium order set quality score is associated with a decreased duration of mechanical ventilation in cardiovascular surgery patients.[J]. Crit Care Med, 2013, 41(11):2610-2617.
24. Hope AA, Morrison RS, Du Q, et al. Risk factors for long-term brain dysfunction after chronic critical illness.[J]. Ann Am Thorac Soc, 2013, 10(4):315-323.
25. Patel SB, Poston JT, Pohlman A, et al. Rapidly reversible, sedation-related delirium versus persistent delirium in the intensive care unit.[J]. Am J Respir Crit Care Med, 2014, 189(6):658-665.
26. Brummel NE, Jackson JC, Pandharipande PP, et al. Delirium in the ICU and subsequent long-term disability among survivors of mechanical ventilation.[J]. Crit Care Med, 2014, 42(2):369-377.
27. Bryczkowski SB, Lopreiato MC, Yonclas PP, et al. Delirium prevention program in the surgical intensive care unit improved the outcomes of older adults.[J]. J Surg Res, 2014, 190(1):280-288.
28. Mehta S, Cook D, Devlin JW, et al. Prevalence, risk factors, and outcomes of delirium in mechanically ventilated adults.[J]. Crit Care Med, 2015, 43(3):557-566.
29. Klompas M, Anderson D, Trick W, et al. The preventability of ventilator-associated events. The CDC Prevention Epicenters Wake Up and Breathe Collaborative.[J]. Am J Respir Crit Care Med, 2015, 191(3):292-301.
30. Hsieh SJ, Soto GJ, Hope AA, et al. The association between acute respiratory distress syndrome, delirium, and in-hospital mortality in intensive care unit patients.[J]. Am J Respir Crit Care Med, 2015, 191(1):71-78.
31. Reade MC, Eastwood GM, Bellomo R, et al. Effect of Dexmedetomidine Added to Standard Care on Ventilator-Free Time in Patients With Agitated Delirium: A Randomized Clinical Trial.[J]. JAMA, 2016, 315(14):1460-1468.
32. Kenes MT, Stollings JL, Wang L, et al. Persistence of Delirium after Cessation of Sedatives and Analgesics and Impact on Clinical Outcomes in Critically Ill Patients.[J]. Pharmacotherapy, 2017, 37(11):1357-1365.
33. Mesa P, Previgliano IJ, Altez S, et al. Delirium in a Latin American intensive care unit. A prospective cohort study of mechanically ventilated patients.[J]. Rev Bras Ter Intensiva, 2017, ;29(3):337-345.
34. Shehabi Y, Bellomo R, Kadiman S, et al. Sedation Intensity in the First 48 Hours of Mechanical Ventilation and 180-Day Mortality: A Multinational Prospective Longitudinal Cohort Study.[J]. Crit Care Med, 2018, 46(6):850-859.
35. Nunes SL, Forsberg S, Blomqvist H, et al. Effect of Sedation Regimen on Weaning from Mechanical Ventilation in the Intensive Care Unit.[J]. Clin Drug Investig, 2018, 38(6):535-543.
36. Shehabi Y, Howe BD, Bellomo R, et al. Early Sedation with Dexmedetomidine in Critically Ill Patients.[J]. N Engl J Med, 2019, 380(26):2506-2517.
37. Garber PM, Droege CA, Carter KE, et al. Continuous Infusion Ketamine for Adjunctive Analgosedation in Mechanically Ventilated, Critically Ill Patients.[J]. Pharmacotherapy, 2019, 39(3):288-296.
38. Ferraioli D, Ferguson L, Carberry M. Quality improvement project aimed at improving the reliability of spontaneous awakening trials in a district general intensive care unit.[J]. BMJ Open Qual, 2019, 8(2):e000518.
39. Brophy A, Cardinale M, Andrews LB, et al. Prospective Observational Evaluation of Sedation and Pain Management Guideline Adherence Across New Jersey Intensive Care Units.[J]. J Pharm Pract, 2019, 32(5):529-533.
40. Smith SE, Hamblin SE, Dennis BM. Effect of Neuromuscular Blocking Agents on Sedation Requirements in Trauma Patients with an Open Abdomen.[J]. Pharmacotherapy, 2019, 39(3):271-279.
41. Khan RM, Al-Juaid M, Al-Mutairi H, et al. Implementing the comprehensive unit-based safety program model to improve the management of mechanically ventilated patients in Saudi Arabia.[J]. Am J Infect Control, 2019, 47(1):51-58.

Sedation depth not report

1. Zappetti D. Does a Sedation Strategy Including Dexmedetomidine Decrease Mortality and the Need for Mechanical Ventilation in Sepsis?[J]. Clin Pulm Med, 2017, 24(4):180.
2. Ruokonen E, Parviainen I, Jakob SM, et al. [Dexmedetomidine versus propofol/midazolam for long-term sedation during mechanical ventilation](https://www-embase-com.ezproxy.lb.polyu.edu.hk/a/#/search/results?subaction=viewrecord&rid=108&page=1&id=L50271983).[J]. Intensive Care Med, 2009, 35(2):282-290.
3. Becker SE. A Pilot Study Implementing a Protocol Using Dexmedetomidine as a Safe Alternative to Traditional Sedation to Decrease Ventilator Days for Patients Difficult to Extubate.[J]. Dimens Crit Care Nurs, 2016, 35(5):291-297.
4. Ely EW, Truman B, Shintani A, et al. Monitoring sedation status overtime in ICU patients: reliability and validity of the Richmond Agitation-Sedation Scale (RASS).[J]. JAMA, 2003, 289(22):2983-2991.
5. de Wit M, Pedram S, Best AM, et al. Observational study of patient-ventilator asynchrony and relationship to sedation level.[J]. J Crit Care, 2009, 24(1):74-80.
6. Carrothers KM, Barr J, Spurlock B, et al. Contextual issues influencing implementation and outcomes associated with an integrated approach to managing pain, agitation, and delirium in adult ICUs[J]. Care Med, 2013, 41(9 Suppl 1):S128-135.
7. Micek ST, Anand NJ, Laible BR, et al. Delirium as detected by the CAM-ICU predicts restraint use among mechanically ventilated medical patients.[J]. Crit Care Med, 2005, 33(6):1260-1265.
8. Robinson BR, Mueller EW, Henson K, et al. An analgesia-delirium-sedation protocol for critically ill trauma patients reduces ventilator days and hospital length of stay.[J]. J Trauma, 2008, 65(3):517-526.
9. Wan RY, Kasliwal M, McKenzie CA, et al. Quetiapine in refractory hyperactive and mixed intensive care delirium: a case series.[J]. Crit Care, 2011, 28;15(3):R159.
10. Mansouri P, Javadpour S, Zand F, et al. Implementation of a protocol for integrated management of pain, agitation, and delirium can improve clinical outcomes in the intensive care unit: a randomized clinical trial.[J]. J Crit Care, 2013, 28(6):918-922.
11. Wanat M, Fitousis K, Boston F, et al. Comparison of dexmedetomidine versus propofol for sedation in mechanically ventilated patients after cardiovascular surgery.[J]. Methodist Debakey Cardiovasc J, 2014, 10(2):111-117.
12. Burry L, Cook D, Herridge M, et al. Recall of ICU Stay in Patients Managed With a Sedation Protocol or a Sedation Protocol With Daily Interruption.[J]. Crit Care Med, 2015, 43(10):2180-2190.
13. MacLaren R, Preslaski CR, Mueller SW, et al. A randomized, double-blind pilot study of dexmedetomidine versus midazolam for intensive care unit sedation: patient recall of their experiences and short-term psychological outcomes.[J]. J Intensive Care Med, 2015, 30(3):167-175.
14. Rivosecchi RM, Kane-Gill SL, Svec S, et al. The implementation of a non-pharmacologic protocol to prevent intensive care delirium.[J]. J Crit Care, 2016, 31(1):206-211.
15. Liu D, Lyu J, Zhao H, et al. The influence of analgesic-based sedation protocols on delirium and outcomes in critically ill patients: A randomized controlled trial.[J]. PLoS One, 2017, 12(9):e0184310.
16. Jablonski J, Gray J, Miano T, et al. Pain, Agitation, and Delirium Guidelines: Interprofessional Perspectives to Translate the Evidence.[J]. Dimens Crit Care Nurs, 2017, 36(3):164-173.
17. Perbet S, Verdonk F, Godet T, et al. Low doses of ketamine reduce delirium but not opiate consumption in mechanically ventilated and sedated ICU patients: A randomised double-blind control trial.[J]. Anaesth Crit Care Pain Med, 2018, 37(6):589-595.
18. Trogrlic Z, van der Jagt M, Lingsma H, et al. Improved Guideline Adherence and Reduced Brain Dysfunction After a Multicenter Multifaceted Implementation of ICU Delirium Guidelines in 3,930 Patients.[J]. Crit Care Med, 2019, 47(3):419-427.
19. Dres M, Younes M, Rittayamai N, et al. Sleep and Pathological Wakefulness at the Time of Liberation from Mechanical Ventilation (SLEEWE). A Prospective Multicenter Physiological Study.[J]. Am J Respir Crit Care Med, 2019, 199(9):1106-1115.
20. Chuich T, Cropsey CL, Shi Y, et al. Perioperative Sedation in Mechanically Ventilated Cardiac Surgery Patients With Dexmedetomidine-Based Versus Propofol-Based Regimens.[J]. Ann Pharmacother, 2019, 53(1):5-12.
21. Wit MD, Gennings C, Jenvey WI, et al. Randomized trial comparing daily interruption of sedation and nursing-implemented sedation algorithm in medical intensive care unit patients.[J]. Critical Care, 2008, 12(3):R70.
22. Elgebaly AS, Sabry M. Sedation effects by dexmedetomidine versus propofol in decreasing duration of mechanical ventilation after open heart surgery.[J]. Ann Card Anaesth, 2018, 21(3):235-242.

Abstract/review/protocol

1. Fan E, Shahid S, Kondreddi VP, et al. Informed consent in the critically ill: a two-step approach incorporating delirium screening.[J]. Crit Care Med, 2008, 36(1):94-99.
2. Toft P, Olsen HT, [Jørgensen](https://pubmed.ncbi.nlm.nih.gov/?term=J%C3%B8rgensen+HK&cauthor_id=25528350) HK, et al. Non-sedation versus sedation with a daily wake-up trial in critically ill patients receiving mechanical ventilation (NONSEDA Trial): study protocol for a randomised controlled trial.[J]. Trials, 2014, 15:499.
3. Strøm T, Toft P. Sedation and analgesia in mechanical ventilation.[J]. Semin Respir Crit Care Med, 2014, 35(4):441-450.
4. Jerath A, Ferguson ND, Steel A, et al. The use of volatile anesthetic agents for long-term critical care sedation (VALTS): study protocol for a pilot randomized controlled trial.[J]. Trials, 2015, 16(1):560.
5. Chen L, Lim FA. Stuck Inside a Cloud: Optimizing Sedation to Reduce ICU-Associated Delirium in Geriatric Patients.[J]. Crit Care Nurs Q, 2015, 38(3):245-252.
6. Nedergaard HK, Jensen HI, Stylsvig M, et al. Non-sedation versus sedation with a daily wake-up trial in critically ill patients receiving mechanical ventilation- effects on long-term cognitive function: study protocol for a randomized controlled trial, a substudy of the NONSEDA trial.[J]. Trials, 2016, 17(1):269.
7. Khan SH, Wang S, Harrawood A, et al. Decreasing Delirium through Music (DDM) in critically ill, mechanically ventilated patients in the intensive care unit: study protocol for a pilot randomized controlled trial.[J]. Trials, 2017, 18(1):574.
8. Schickli MA, Eberwein KA, Short MR, et al. Pharmacy-Driven Dexmedetomidine Stewardship and Appropriate Use Guidelines in a Community Hospital Setting.[J]. Ann Pharmacother, 2017, 51(1):27-32.
9. Fuller BM, Mohr NM, Roberts BW, et al. Protocol for a multicentre, prospective cohort study of practice patterns and clinical outcomes associated with emergency department sedation for mechanically ventilated patients: the ED-SED Study.[J]. BMJ Open., 2018, 8(10):e023423.
10. Nedergarrd HK, Jensen HI, Stylsvig M, et al. [Effect of non-sedation on cognitive function in survivors of critical illness](https://www-embase-com.ezproxy.lb.polyu.edu.hk/a/#/search/results?subaction=viewrecord&rid=1&page=1&id=L631811600).[J]. Intensive Care Med Exp, 2019, 7(Suppl 3).
11. Walczak KD, Otero CV, Grewal D, et al. [Impact of volatile anesthetics for long-term sedation in critically ill patients on cognitive impairment at 3-months follow-up](https://www-embase-com.ezproxy.lb.polyu.edu.hk/a/#/search/results?subaction=viewrecord&rid=7&page=1&id=L630354111).[J]. Am J Respir Crit Care Med, 2019, 199(9).
12. Takoua K, Aymen M, Nasreddine F, et al. [The influence of sedation choice on the delirium occurrence in critically ill poisoned patients: A randomized controlled trial](https://www-embase-com.ezproxy.lb.polyu.edu.hk/a/#/search/results?subaction=viewrecord&rid=23&page=1&id=L620836855).[J]. Ann Intensive Care, 2018, 8(Suppl 1).
13. Ali D, Clark J, Flannery A, et al. [De-escalation of dexmedetomidinebased sedation utilizing clonidine in medical and surgery icus](https://www-embase-com.ezproxy.lb.polyu.edu.hk/a/#/search/results?subaction=viewrecord&rid=25&page=1&id=L620080156).[J]. Crit Care Med, 2018, 46(Suppl 1):489.
14. Wong A, Derry K, Namba J, et al. [A retrospective comparison of safety and efficacy of dexmedetomidine to propofol beyond 120 hours](https://www-embase-com.ezproxy.lb.polyu.edu.hk/a/#/search/results?subaction=viewrecord&rid=28&page=1&id=L620080412).[J]. Crit Care Med, 2018, 46(Suppl 1):463.
15. Louie J, Lonardo N, Mone M, et al. [Does the addition of dexmedetomidine to propofol sedation reduce duration of mechanical ventilation?](https://www-embase-com.ezproxy.lb.polyu.edu.hk/a/#/search/results?subaction=viewrecord&rid=31&page=1&id=L620080711)[J]. Crit Care Med, 2018, 46(Suppl 1):772.
16. Wolf L, Messana E, Wilson SS, et al. [Impact of intermittent versus continuous infusion of fentanyl after rapid sequence intubation on intensive care unit delirium](https://www-embase-com.ezproxy.lb.polyu.edu.hk/a/#/search/results?subaction=viewrecord&rid=38&page=1&id=L620857847).[J]. Ann Emerg Med, 2017, 4(Suppl 1):S37.
17. Strøm T, Olsen H, Nedergaard H, et al. [No sedation for critically ill patients undergoing mechanical ventilation: A multicenter trial](https://www-embase-com.ezproxy.lb.polyu.edu.hk/a/#/search/results?subaction=viewrecord&rid=48&page=1&id=L613520743).[J]. Crit Care Med, 2016, 44(Suppl 1):378.
18. Franco E, Mak G, Cortes J.[Duration of mechanical ventilation with nonbenzodiazepine versus benzodiazepine-based sedation](https://www-embase-com.ezproxy.lb.polyu.edu.hk/a/#/search/results?subaction=viewrecord&rid=51&page=1&id=L613522723).[J]. Crit Care Med, 2016, 44(Suppl 1):323.
19. Lizza B, Jagow B, Hensler D, [Impact of multiple daily clinical pharmacist-enforced assessments on time in target sedation range](https://www-embase-com.ezproxy.lb.polyu.edu.hk/a/#/search/results?subaction=viewrecord&rid=52&page=1&id=L613522802).[J]. Crit Care Med, 2016, 44(Suppl 1):326.
20. Trogrlic Z, Van Der Jagt M, Lingsma H, et al. [Impact of a tailored multifaceted implementation of the pain, agitation and delirium guidelines in adult critically ill patients on guideline adherence, delirium and clinical outcomes: A prospective multicenter before-after study](https://www-embase-com.ezproxy.lb.polyu.edu.hk/a/#/search/results?subaction=viewrecord&rid=59&page=1&id=L617955599).[J]. Intensive Care Med Exp, 2016, 4(Suppl 1).
21. Myatra SN, Harish MM, Prabu NR, et al. [Prospective study to determine the incidence and risk factors for delirium in cancer patients in ICU](https://www-embase-com.ezproxy.lb.polyu.edu.hk/a/#/search/results?subaction=viewrecord&rid=61&page=1&id=L618007791).[J]. Intensive Care Med Exp, 2016, 4(Suppl 1).
22. Contreras RS, Toapanta ND, Moreno G, et al. [A pilot study using bispectral index (BIS) to adjust sedation in patients with no neurological pathology in elderly critical care patients](https://www-embase-com.ezproxy.lb.polyu.edu.hk/a/#/search/results?subaction=viewrecord&rid=62&page=1&id=L618007846).[J]. Intensive Care Med Exp, 2016, 4(Suppl 1).
23. Miyamoto K, Kawazoe Y, Morimoto T, et al. [Dexmedetomidine for ventilated septic patients in ICU: A multicenter randomized controlled trial](https://www-embase-com.ezproxy.lb.polyu.edu.hk/a/#/search/results?subaction=viewrecord&rid=63&page=1&id=L618008012).[J]. Intensive Care Med Exp, 2016, 4(Suppl 1).
24. Ramya K, Kumar V, Rao R, et al. [Melatonin to prevent delirium in organophosphorus compound poisoning patients: A double blind randomised control trial](https://www-embase-com.ezproxy.lb.polyu.edu.hk/a/#/search/results?subaction=viewrecord&rid=80&page=1&id=L624508679).[J].Indian J Crit Care Med, 2015, 19(Suppl 1):S8-S9.
25. Singh A, Ambike D, Thatte WS, et al. [Dexmedetomidine versus midazolam infusion for sedation in mechanically ventilated patients in critical care setting: A randomized controlled trial](https://www-embase-com.ezproxy.lb.polyu.edu.hk/a/#/search/results?subaction=viewrecord&rid=92&page=1&id=L71239307).[J]. Indian J Crit Care Med, 2013, 17(Suppl 2):4.
26. Shehabi Y, Bellomo R, Reade M, et al.[Early goal directed sedation vs standard care sedation in mechanically ventilated critically ill adults; a randomized controlled pilot trial](https://www-embase-com.ezproxy.lb.polyu.edu.hk/a/#/search/results?subaction=viewrecord&rid=94&page=1&id=L71065239).[J]. Crit Care Med Exp, 2012, 40(Suppl 1):16.

Outcomes data lacking

1. Pun BT, Gordon SM, Peterson JF, et al. Large-scale implementation of sedation and delirium monitoring in the intensive care unit: a report from two medical centers.[J]. Crit Care Med, 2005, 33(6):1199-1205.
2. Schweickert WD, Pohlman MC, Pohlman AS, et al. Early physical and occupational therapy in mechanically ventilated, critically ill patients: a randomised controlled trial.[J]. Lancet, 2009, 373(9678):1874-1882.
3. Needham DM, Korupolu R, Zanni JM, et al. Early physical medicine and rehabilitation for patients with acute respiratory failure: a quality improvement project.[J]. Arch Phys Med Rehabil, 2010, 91(4):536-542.
4. Trouillet JL, Luyt CE, Guiguet M, et al. Early percutaneous tracheotomy versus prolonged intubation of mechanically ventilated patients after cardiac surgery: a randomized trial.[J]. Ann Intern Med, 2011, 154(6):373-383.
5. Strøm T. Sedation in the ICU.[J]. Dan Med J, 2012, 59(5):B4458.
6. Shehabi Y, Chan L, Kadiman S, et al. Sedation depth and long-term mortality in mechanically ventilated critically ill adults: a prospective longitudinal multicentre cohort study.[J]. Intensive Care Med, 2013, 39(5):910-918.
7. Tedders KM, McNorton KN, Edwin SB. Efficacy and safety of analgosedation with fentanyl compared with traditional sedation with propofol.[J]. Pharmacotherapy, 2014, 34(6):643-647.
8. Faust AC, Rajan P, Sheperd LA, et al. Impact of an Analgesia-Based Sedation Protocol on Mechanically Ventilated Patients in a Medical Intensive Care Unit.[J]. Anesth Analg, 2016, 123(4):903-909.
9. Humphrey M, Everhart S, Kosmisky D, et al. An evaluation of patient-specific characteristics on attainment of target sedation in an intensive care unit.[J]. Heart Lung, 2018, 47(4):387-391.
10. Mistraletti G, Umbrello M, Salini S, et al. Enteral versus intravenous approach for the sedation of critically ill patients: a randomized and controlled trial.[J]. Crit Care, 2019, 23(1):3.

No english

1. Hu Z., Zhang ZC, Li D, et al. [The use of dexmedetomidine combined with propofol in mechanically ventilated patients](https://www-embase-com.ezproxy.lb.polyu.edu.hk/a/#/search/results?subaction=viewrecord&rid=82&page=1&id=L605046941).[J].Med J Chin Peoples Liberation Army, 2015, 40(6):479-483.
2. [Pérez-Rada](https://pubmed.ncbi.nlm.nih.gov/?term=P%C3%A9rez-Rada+Fde+J&cauthor_id=23234746) J, [Macías-García](https://pubmed.ncbi.nlm.nih.gov/?term=Mac%C3%ADas-Garc%C3%ADa+MT&cauthor_id=23234746) MT, Cataneo-Cerna AC. Postsurgical delirium complicated with sepsis. Dexmedetomidine versus midazolam.[J]. Rev Med Inst Mex Seguro Soc, 2012, 50(4):419-426.
3. Volz D, Vogt A, [Schütz](https://pubmed.ncbi.nlm.nih.gov/?term=Sch%C3%BCtz+M&cauthor_id=24820355) M, et al. Methohexital for analgosedation of ventilated intensive care patients : prospective nonrandomized single center observational study on incidence of delirium.[J]. Anaesthesist, 2014, 63(6):488-495.
4. Nikoda VV, Gritsan AI, Eremenko AA, et al. The efficacy and safety of dexmedetomidine for sedation of patients during prolonged mechanical ventilation in intensive care units( Russian multicenter study results ).[J]. Anesteziol Reanimatol, 2015, 60(5):47-53.
5. Guo K, Zhang H, Peng S. Comparison of two schemes of daily arousal and comfort analgesia and sedation in patients on mechanical ventilation in intensive care unit.[J]. Zhonghua Wei Zhong Bing Ji Jiu Yi Xue, 2018, 30(10):950-952.
6. Zhang M, Yuan J, Chen Q, et al. Application of Narcotrend index and Richmond agitation-sedation score in sedation assessment of patients with short-term mechanical ventilation after pancreatoduodenectomy: a randomized controlled trial.[J]. Zhonghua Wei Zhong Bing Ji Jiu Yi Xue, 2019, 31(6):737-741.

**STEP 4: 15 studies included after full text review and 3 studies included from second search**

1. Nassar Junior AP, Park M. Daily sedative interruption versus intermittent sedation in mechanically ventilated critically ill patients: a randomized trial. Ann Intensive Care, 2014, 4(1):14.
2. Pandharipande PP, Pun BT, Herr DL, et al. Effect of sedation with dexmedetomidine vs lorazepam on acute brain dysfunction in mechanically ventilated patients: the MENDS randomized controlled trial.[J]. JAMA, 2007, 298(22):2644-2654.
3. Strøm T, Martinussen T, Toft P. A protocol of no sedation for critically ill patients receiving mechanical ventilation: a randomized trial.[J]. Lancet, 2010, 375(9713):475-480.
4. Shehabi Y, Bellomo R, Reade MC, et al. Early intensive care sedation predicts long-term mortality in ventilated critically ill patients.[J]. Am J Respir Crit Care Med, 2012, 186(8):724-731.
5. Shehabi Y, Bellomo R, Reade MC, et al. Early goal-directed sedation versus standard sedation in mechanically ventilated critically ill patients: a pilot study*.[J]. Crit Care Med, 2013, 41(8):1983-1991.
6. Dale CR, Kannas DA, Fan VS, et al. Improved analgesia, sedation, and delirium protocol associated with decreased duration of delirium and mechanical ventilation.[J]. Ann Am Thorac Soc, 2014, 11(3):367-374.
7. Skrupky LP, Drewry AM, Wessman B, et al. Clinical effectiveness of a sedation protocol minimizing benzodiazepine infusions and favoring early dexmedetomidine: a before-after study.[J]. Crit Care, 2015, 19(1):136.
8. Kawazoe Y, Miyamoto K, Morimoto T, et al. Effect of Dexmedetomidine on Mortality and Ventilator-Free Days in Patients Requiring Mechanical Ventilation With Sepsis: A Randomized Clinical Trial.[J]. JAMA, 2017, 317(13):1321-1328.
9. Kaplan JB, Eiferman DS, Porter K, et al. Impact of a nursing-driven sedation protocol with criteria for infusion initiation in the surgical intensive care unit.[J]. J Crit Care, 2018, 30(50):195-200.
10. Samuelson KAM, Lundberg D, Fridlund B. Light vs. heavy sedation during mechanical ventilation after oesophagectomy–a pilot experimental study focusing on memory. Acta Anaesthesiol Scand, 2008, 52(8):1116-1123.
11. Treggiari MM, Romand JA, Yanez ND, et al. Randomized trial of light versus deep sedation on mental health after critical illness*. Crit Care Med, 2009, 37(9):2527-2534.
12. Girard TD. Efficacy and safety of a paired sedation and ventilator weaning protocol for mechanically ventilated patients in intensive care (Awakening and Breathing Controlled trial): a randomised controlled trial. Lancet, 2008, 371(9607):126-134.
13. van den Boogaard M, Schoonhoven L, van der Hoeven JG, et al. Incidence and short-term consequences of delirium in critically ill patients: A prospective observational cohort study. Int J Nurs Stud, 2012, 49(7):775-783.
14. Hager DN, Dinglas VD, Subhas S, et al. Reducing Deep Sedation and Delirium in Acute Lung Injury Patients: A Quality Improvement Project*. Crit Care Med, 2013, 41(6):1435-1442.
15. Shehabi Y, Ismail WN, Saman MA, et al. Early goal directed sedation with dexmedetomidine vs standard sedatives, randomized control trial. Crit Care Med, 2013, 41(2):A217.
16. Balzer F, Weib B, Kumpf O, et al. Early deep sedation is associated with decreased in-hospital and two-year follow-up survival. Crit Care, 2015, 19(1):197.
17. Stephens RJ, Ablordeppey E, Drewry AM, et al. Analgosedation practices and the impact of sedation depth on clinical outcomes among patients requiring mechanical ventilation in the emergency department: a cohort study. Chest, 2017, 152(5):963-971.
18. De Jonghe B, Aboab J, Aissaoui N, et al. Impact of oversedation prevention in ventilated critically ill patients: a randomized trial-the AWARE study. Ann Intensive Care, 2018, 8(1):93.
