# Supplementary material for: Different depths of sedation versus risk of delirium in adult mechanically ventilated patients: A systematic review and meta-analysis
Source: PLoS One. 2020 Jul 16;15(7):e0236014. doi: 10.1371/journal.pone.0236014 (PMC7365415; doi:10.1371/journal.pone.0236014)
Supplement: Attachment — Submitted filename: Response to Reviewers.docx [file pone.0236014.s009.docx]

Dear Editors and Reviewers:

Thank you for letter and for the reviewer’ comments concerning our manuscript entitled “Different Depths of Sedation Versus Risk of Delirium in Adult Mechanically Ventilated Patients: A Systematic Review and Meta-Analysis”(ID: PONE-D-19-33669). Those comments are all valuable and very helpful for revising and improving our paper, as well as the important guiding significance to our research. We have read the comments carefully, have discussed and made correction with all authors. We hope the revised version meet with approval. The main corrections in the paper and the responds to the reviewer’s comments are listed below.

Part I: Journal Requirements

Q1 When submitting your revision, we need you to address these additional requirements.
Please ensure that your manuscript meets PLOS ONE's style requirements, including those for file naming. The PLOS ONE style templates can be found at <http://www.journals.plos.org/plosone/s/file?id=wjVg/PLOSOne_formatting_sample_main_body.pdf> and <http://www.journals.plos.org/plosone/s/file?id=ba62/PLOSOne_formatting_sample_title_authors_affiliations.pdf>

**Response:** The submitted documents(Revised Manuscript with Track Changes) have been modified as much as possible according to the journal requirements, and some of the revised portion are marked in yellow in the paper.

Q2 We note that the original search was performed in December 2019. To ensure that your systematic review is up-to-date and comprehensive, we ask that you please update the search period to ensure that the literature search includes studies that have been published within the last 12 months.

**Response:** We have updated the search period to ensure the literatures that published within the last 12 months were included. But no new study was included. The revised portion as shown on line 18, 61 to 63, 127 to 129, marked in red.

Q3 At this time, we ask that you please move the Newastle-Ottawa scale quality assessment, risk of bias graph and funnel plot provided as additional files in your submission to the manuscript as Figure files.

**Response:** We have upload the risk of bias graph and funnel plot as Figure files.

Q4 PLOS requires an ORCID iD for the corresponding author in Editorial Manager on papers submitted after December 6th, 2016. Please ensure that you have an ORCID iD and that it is validated in Editorial Manager. To do this, go to ‘Update my Information’ (in the upper left-hand corner of the main menu), and click on the Fetch/Validate link next to the ORCID field. This will take you to the ORCID site and allow you to create a new iD or authenticate a pre-existing iD in Editorial Manager. Please see the following video for instructions on linking an ORCID iD to your Editorial Manager account: https://www.youtube.com/watch?v=_xcclfuvtxQ

**Response:** I have an ORCID iD and update my information.

5.Please include your tables as part of your main manuscript and remove the individual files. Please note that supplementary tables (should remain/ be uploaded) as separate "supporting information" files.

**Response:** We have made the table 1 as part of manuscript and removed the individual files.

6. Thank you for stating the following financial disclosure:

a) Please provide an amended Funding Statement that declares *all* the funding or sources of support received during this specific study (whether external or internal to your organization) as detailed online in our guide for authors at <http://journals.plos.org/plosone/s/submit-now>.

b) Please state what role the funders took in the study.  If any authors received a salary from any of your funders, please state which authors and which funder. If the funders had no role, please state: "The funders had no role in study design, data collection and analysis, decision to publish, or preparation of the manuscript."

Please include your amended statements within your cover letter; we will change the online submission form on your behalf.

**Response:** There are no funding that was stated in our cover letter.

7. Thank you for stating the following in your Competing Interests section:

Please complete your Competing Interests on the online submission form to state any Competing Interests. If you have no competing interests, please state "The authors have declared that no competing interests exist.", as detailed online in our guide for authors at <http://journals.plos.org/plosone/s/submit-now>

This information should be included in your cover letter; we will change the online submission form on your behalf.

**Response**: The information has been included in our cover letter.

Part II: Additional Editor Comments

Q My assessment is very similar to the two reviewers. I think that paper is described but it needs to add some important analyzes, for example the quality analysis of the body of evidence separately for each outcome (GRADE) as mentioned by one of the reviewers. I also didn't see the discussion about sensitivity and heterogeneity analysis. I also agree with the lack of clarity about the exposure variable definition of depth of sedation with different cut-offs for sedation depths among the included studies. It would be great if authors could answer point by point suggested by reviewers.

**Response**: Thank you for your comments. The quality analysis of the body of evidence separately for each outcome(GRADE) has been uploaded as S2 File.

The sensitivity and heterogeneity have been analyzed in the last paragraph of the disscussion. A high bias risk was not identified by sensitivity analysis. The source of heterogeneity may be related to the type of included studies. The heterogeneity is obviously reduced in the subgroup analysis, which showed the results consistent with the overall results.

You mentioned the lack of clarity about the exposure variable definition of sedation depth with different cut-offs for sedation depths among the included studies. It is true that the definition regarding the depth of sedation such as light or deep sedation differed among the selected studies for this meta-analysis. This was the reason that the definition of “lighter sedation” vs “deeper sedation” was used rather than “light sedation” vs “deep sedation” in our study, targeting the effect resulted from different sedation depths. So, the specific cut-off of sedation wasn’t emphasized.

Part III: Reviewers' comments

Reviewer #1

Q1 I am a little concerned about the exposure variable definition of depth of sedation. As different studies used different cut-offs for sedation depths, sweeping statement based on combining these different exposures may lead us to inconclusive findings as seen in the review. That will be my major concern while trying to conclude depths of sedation as a risk factor for delirium when the exposure depths are not same in the studies. While most studies seem to be using the cut-off of RASS greater than or equal to -3, it is not the same with all the studies. It may be worthwhile to pool studies which are consistent and see if the findings hold.

**Response:** You mentioned the lack of clarity about the exposure variable definition of sedation depth with different cut-offs for sedation depths among the included studies. It is true that the definition regarding the depth of sedation such as light or deep sedation differed among the selected studies for this meta-analysis. This was the reason that the definition of “lighter sedation” vs “deeper sedation” was used rather than “light sedation” vs “deep sedation” in our study, targeting the effect resulted from different sedation depths. So, the specific cut-off of sedation wasn’t emphasized. The intervention of some studies in our review is not sedation depth difference, but sedation depth difference was found in the research process. We need high-quality study(grouped by specific sedation depth) to verify this finding and our team has been working for this.

Q2 The other major concern I have is regarding the outcome variable which is delirium incidence. How is this defined? In the literature, it has been defined multiple ways and I am not sure how the authors have construed it for their review. This again will result in discrepancy among studies. As there has been debate about how to define incidence versus prevalence especially in ICU settings where patients may have been admitted with delirium, the authors may want to think this over. One suggestion is to keep it general with a term such as delirium occurrence.

**Response:** Considering the reviewer’s suggestion, we have used delirium occurrence instead of delirium incidence. Regard to the factors may confound the evaluation of delirium, patients with delirium delirium before enrollment were excluded.

Q3 An interesting finding to note is the absence of a confounding effect of BZD on delirium. This really needs to be highlighted in the discussion. BZD have been thought of as risk factors for delirium, but the authors findings point towards a much-nuanced issue. Are these effects independent of sedation depth or is it a direct effect? The authors need to comment further on this in the discussion.

**Response:** Our study found there is no significant difference in the delirium risk between different sedation depths among studies showing a significant difference in the dosage of benzodiazepines. Maybe these effects are not independent of sedation depth, because the lighter sedation group had a lower dosage of benzodiazepines.

Q4 The findings of light sedation increasing delirium risk need to be further elaborated as this seems counter intuitive to current approaches.

**Response:** The use of sedatives is important for ensuring comfort and safety, especially during the mechanical ventilation phase. Deep levels of sedation maybe protect patients from experiencing discomfort and reduce patients’ stressful memories. A cohort study found that patients with lighter sedation were more likely to remember the painful experience of ICU after discharge.

Q5 Based on the mortality findings, in the absence of increasing delirium risk, depth of sedation is independently associated with mortality. Is this cause or could this be due to sicker patients kept deeply sedated. I would suspect that as this finding holds in RCT with randomized approach, the latter is not the case. This will need further discussion in the discussion section. May be an analysis with stratification based on illness severity may help clarify this further.

**Response:** Our study showed that depth of sedation is independently associated with mortality. According to your comments, we conducted a subgroup analysis on whether there are differences in disease severity. For 11 studies that disease severity did not differ, the meta-analysis showed that the deeper sedation had a significantly higher risk of mortality(OR=1.20, 95%CI: 1.03-1.39, *P*=0.020). The other 5 studies also showed the deeper sedation had a significantly higher risk of mortality(OR=3.95, 95%CI: 2.67-5.86, *P*=0.000).

Reviewer #2

Q Although the authors did not assess the quality of the evidence set and the strength of the recommendation (GRADE), for each of the results analyzed, I think that the review was very well conducted, that the problem of delusion is the order of the day in intensive care units and, why, the results of the studies may help a lot in making decisions about sedation and analgesia of patients in these units.

**Response:** The quality analysis of the body of evidence separately for each outcome(GRADE) has been uploaded as S2 File.

We tried our best to improve the manuscript and made some changes in the manuscript. But these changes will not influence the content and framework of the paper. We appreciate for Editors/Reviewers’ warm work, and hope that the correction will meet with approval. If you have any questions, please contact us without hesitate. Once again, thank you very much for your comments and suggestions.

Sincerely

Ling Long, Heling Zhao, Penglin Ma, et al.
