# Supplementary material for: Different depths of sedation versus risk of delirium in adult mechanically ventilated patients: A systematic review and meta-analysis
Source: PLoS One. 2020 Jul 16;15(7):e0236014. doi: 10.1371/journal.pone.0236014 (PMC7365415; doi:10.1371/journal.pone.0236014)
Supplement: Attachment — Submitted filename: Response to Reviewers(R2).docx [file pone.0236014.s010.docx]

Dear Editors and Reviewers:

Thank you for letter and for the reviewer’ comments concerning our manuscript entitled “Different Depths of Sedation Versus Risk of Delirium in Adult Mechanically Ventilated Patients: A Systematic Review and Meta-Analysis”(ID: PONE-D-19-33669). Those comments are all valuable and very helpful for revising and improving our paper. We have discussed and made correction with all authors. We hope the revised version meet with approval.

Reviewers' comments

Reviewer #3

With great interest I read the adapted version of the manuscript. The authors addressed the comments of the previous reviewers adequately which led to significant improvement of the research and the manuscript. The authors did a rigorous meta-analysis and show great adherence to existing standards and protocols in place for such research. Doing so, the research is up to current standards and very suitable for publication in Plos One. And although the manuscript is almost in its final version I do have some concerns reading the research for the first time.
 My main concern is the fact that the relationship between sedation (depth) and delirium is very complex and influenced by many factors. The effort of the authors to try to include observational studies in their meta analysis is a great effort but introduces the bias of observational research in their meta analysis. Observational research in ICU research is prone to bias and relies on advanced statistical methods including time-varying variables, competing events, confounding by indication, and addressing immortal time bias. For example, there is the challenge that light sedation levels interfere with delirium diagnosis and comparing them with deep sedation give therefore regression to the mean (for example patient scoring RASS-2 is CAM-ICU positive (item 1, 2 and 4 present)).Or the problem that disease severity and dosage of sedatives are not pure confounding factors as both cause deep sedation (placing them in causal chain of high severity of illness-->deep sedation-->delirium). This causal chain is also seen in the risk of deep sedation and risk of mortality; deep sedation being a proxy for severity of illness and thus not surprisingly being associated with higher mortality. Or the challenge that only patients in light sedation have the opportunity to develop adverse events (immortal time bias..you have to be awake enough to remove the ET).
 I feel that the authors do address a lot or these issues by reporting the results separately for RCTs and observational research but I would appreciate it if the authors emphasize the risk of bias of observational research and thus of the reported overall ORs. For example, the overall ORs on the relationship of mortality and adverse events seem to be driven by the observational research.

**Response:** Thanks again for your comments. Indeed, the relationship between the depth of sedation and delirium is complex and influenced by many factors. The severity of the disease and the dosage of benzodiazepines are considered to be important factors. Therefore, we conducted a subgroup analysis of these factors and found that differences in the benzodiazepine dosage did not alter the conclusion that sedation depth does not influence the risk of delirium. But the patients with the same severity of the disease have the higher risk of delirium with the lighter sedation(OR = 0.68, 95% CI: 0.49-0.94, *P* = 0.021) and higher risk of mortality with the deeper sedation(OR = 1.20, 95% CI: 1.03-1.39, *P* = 0.020). So, the severity of illness seemed to confound this conclusion less likely.

Our systematic review included some observational research, which generated high heterogeneity. In order to reduce the weight of observational research in the overall results, we used a Bayesian method. It is found that the results of most subgroup analyses seemed similar to those of the general meta-analysis. In terms of the risk of mortality and incidence of adverse events, the ORs of the RCTs, observational research and overall research were all on the same side of the forest plots. Nevertheless, we agree with and appreciate the comments of reviewer #3. We have stressed these views in the discussion of the revised manuscript. "Due to the methodological limitations, we should carefully interpret the meta results of observational research. It is evident that the likelihood of diagnosing delirium and developing adverse events is higher in patients sedated with the lighter depth, while the effect of deep sedation on mortality needs to be adjusted with factors such as disease severity. " (shown on page 13, line 330 to 336, marked in red.)

We tried our best to improve the manuscript. We appreciate for Editors/Reviewers’ warm work, and hope that the correction will meet with approval. If you have any questions, please contact us without hesitate. Once again, thank you very much for your comments and suggestions.

Sincerely

Ling Long, Heling Zhao, Penglin Ma, et al.
