# Supplementary material for: Different depths of sedation versus risk of delirium in adult mechanically ventilated patients: A systematic review and meta-analysis
Source: PLoS One. 2020 Jul 16;15(7):e0236014. doi: 10.1371/journal.pone.0236014 (PMC7365415; doi:10.1371/journal.pone.0236014)
Supplement: Attachment — Submitted filename: Response to Reveiwers(R3).docx [file pone.0236014.s011.docx]

Dear Editors:

Thank you for letter and for the comments concerning our manuscript entitled “Different Depths of Sedation Versus Risk of Delirium in Adult Mechanically Ventilated Patients: A Systematic Review and Meta-Analysis”(ID: PONE-D-19-33669). We have read the comments carefully, have discussed and made correction with all authors. We hope the revised version meet with approval.

Q1: To explain their selection of the study designs for inclusion in the review in method section - Explanation for including both RCTs and NRSI

Response: There is a lack of research published RCTs primarily regarding the effect of sedation depth on delirium occurrence in critically ill patients with MV and more studies on the outcome of delirium are cohort study. Therefore, it is suggested that the inclusion of the cohort study may increase the information of systematic review on the exploration of delirium risk factors, but the lower quality of data is a fact. The RCTs and NRSI must meet the PICO principle. The exclusion criteria have been clarified in the methods section in this paper(marked in blue).

Q2: To provide a list of excluded studies and justify the exclusions

Q3: To report on the sources of funding for the studies included in the review

Response: Thank you for your comments. The list of excluded studies and the sources of funding for the studies included in the review has been uploaded as S3 File and S4 File respectively..

We have tried our best to improve the manuscript and made some changes in the manuscript. We appreciate for Editors’ warm work, and hope that the correction will meet with approval. If you have any questions, please contact us without hesitate. Once again, thank you very much for your comments and suggestions.

Sincerely

Ling Long, Heling Zhao, Penglin Ma, et al.
